# The population genetic structure of *Corythucha ciliata* (Say) (Hemiptera: Tingidae) provides insights into its distribution and invasiveness

**DOI:** 10.1038/s41598-017-00279-5

**Published:** 2017-04-04

**Authors:** Wen-Yan Yang, Xiao-Tian Tang, Rui-Ting Ju, Yong Zhang, Yu-Zhou Du

**Affiliations:** 1 School of Horticulture and Plant Protection & Institute of Applied Entomology, Yangzhou University, Yangzhou, 225009 China; 2 Ministry of Education Key Laboratory for Biodiversity Science and Ecological Engineering, Fudan University, Shanghai, 200438 China; 3 Department of Biology, University of Nevada, Reno, NV USA; 4 Jiangsu Key Laboratory of Crop Genetics and Physiology/Co-Innovation Center for Modern Production Technology of Grain Crops, Yangzhou University, Yangzhou, 225009 China

## Abstract

*Corythucha ciliata* (Say), an invasive pest originating from North America, causes severe damage on sycamore trees. However, little is known about the population genetics and evolutionary forces underlying the invasiveness of this important pest. In the present study, we use three mitochondrial genes (*COI*, *ND1* and *ND5*) and nine microsatellite markers to investigate the population genetics of *C. ciliata* and retrace its spread through China. The results suggest a low level of genetic diversity in Chinese and European populations of *C. ciliata*. Our results indicate that populations of *C. ciliata* have obvious genetic structure, and genetic differentiation is not caused by geographic isolation. In median-joining networks, we observed a higher frequency of shared haplotypes in groups 1 and 3. Based on gene flow and approximate Bayesian computation analyses, we discovered that *C. ciliata* first invaded the east coast of China and subsequently moved inland. Demographic analysis suggested that populations of *C. ciliata* in China may have undergone a recent bottleneck effect. Finally, our results suggest that population structure, high gene flow and environmental conditions have favored the broad invasiveness of this important pest.

## Introduction

The sycamore lace bug, *Corythucha ciliata* (Say) (Hemiptera: Tingidae), is an invasive insect species that originated from the eastern and central regions of North America^1^. It spread to Italy in 1960^2^ and then became widely-distributed throughout Europe^3^. Shortly after 1990, it successively invaded South America^4^, Asia^5^ and Australia^6^. *C*. *ciliata* causes damage to sycamore trees by feeding on the foliage, thus reducing photosynthesis and promoting disease. Once established, it can form fairly stable, high density populations^7–10^. *C*. *ciliata* has become a primary pest of trees in the genus *Platanus* ^11–13^, and effective controls are lacking during widespread outbreaks^14^. For example, *C. ciliata* caused serious damage throughout Europe in the 1980 s, and this resulted in the formation of a special working group to study its biology, ecology, nature enemies and chemical control.

In China, *C*. *ciliata* was first discovered in Changsha in 2002^15^ and then in Wuhan^16, 17^. In a relatively short time, it dispersed to at least 23 cities in China and to the Yangtze River basin^17–20^. Why is *C*. *ciliata* so successful in invading new places? It is helpful to note that this insect has a strong reproductive capacity, and adult females can produce 250–350 eggs^20^. Secondly, *C. ciliata* can withstand both high and low temperature extremes. In laboratory experiments, the Ltemp50 and Ltemp90 of *C. ciliata* adults and nymphs were 41.4 °C and 43.8 °C, respectively, after one hour of high temperature stress^21^. *C. ciliata* adults can also survive after exposure to very low temperatures; e.g. −23.3 °C. Additionally, *C. ciliata* is easily dispersed; although its wings are relatively weak, it can migrate thousands of meters via wind currents^22^ and has been extensively disseminated by human activity^1^. For example, it may have spread from North America to Italy as a result of the shipping industry^10^. In Turkey, *C. ciliata* has dispersed to large cities located along highways^23^, which indicates that human transportation is a major factor in dissemination. Finally, the host trees, *Platanus* spp., are widely planted in urban areas. *C. ciliata* has presumably co-evolved with *Platanus* spp.^24^, which contains seven tree species originating from North America, Europe, and Asia.

Mitochondrial DNA sequences and microsatellites are frequently used to investigate the genetic structure of populations^25–27^, and the combined use of these two genetic markers may result in more accurate information than that obtained from a single marker^28^. In the present study, three mtDNA genes (cytochrome c oxidase subunit I, *COI*), NADH dehydrogenase subunits 1 and 5 (*ND1* and *ND5*) and nine microsatellite loci were used to study the genetic structure of 21 Chinese and two European populations of *C. ciliata*. The primary goals of this study were to: (1) investigate the genetic structure and diversity of *C. ciliata* populations in China and Europe; (2) identify the potential routes of dispersal in China; (3) define the demographic profile associated with the expansion of *C. ciliata*; and (4) analyze the genetic and environmental factors contributing to the invasiveness of *C. ciliata*.

## Results

### Genetic diversity

**Figure 1:**
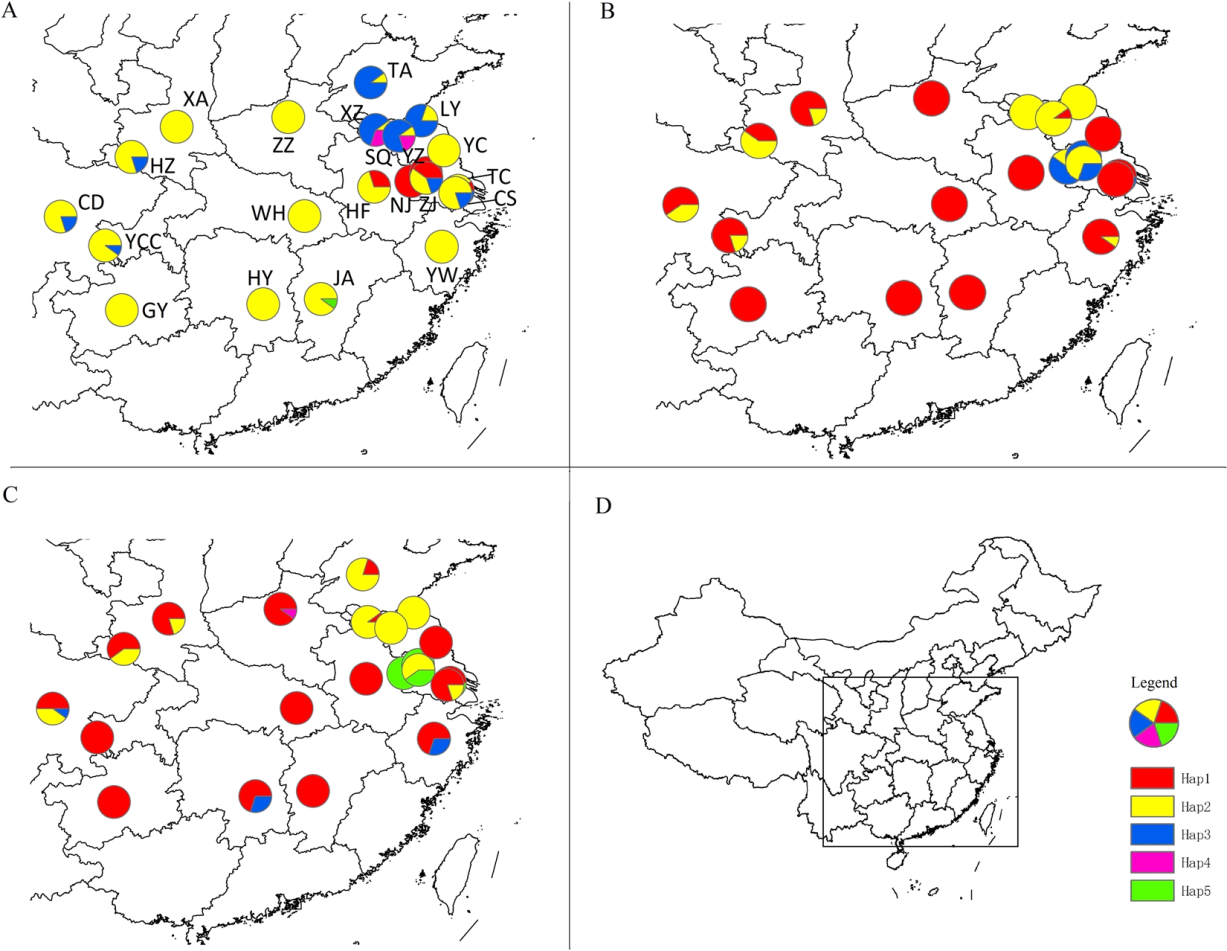
Collection sites in China and haplotype distribution in each population. (**A**,**B** and **C**) represent *COI*, *ND1* and *ND5*, respectively; (**D**) national map and legend. The map is made by ArcGIS 10.2 software, http://www.arcgis.com/features/.

Using the mitochondrial markers, five haplotypes (H1–H5) were identified for *COI*, *ND1* and *ND5* (Fig. 1) (GenBank Accession numbers: KP420509-KP420523). Parameters of genetic diversity were calculated for the 23 populations based on concatenated sequences (Table 1), and *COI*, *ND1*, and *ND5* sequences independently (Supplementary Table S1). The number of haplotypes per population (n) ranged from 1–6, 1–3, 1–2 and 1–3 in concatenated sequences, *COI*, *ND1*, and *ND5*, respectively. Haplotype diversity (H) ranged from 0.000–0.889, 0.000–0.711, 0.000–0.533, and 0.000–0.644 in concatenated sequences, *COI*, *ND1* and *ND5*, respectively. Based on concatenated sequences, the H values of the ZJ and SV populations were the highest (H = 0.889) and the GY, WH, YZ and YC populations were the lowest (H = 0.000) (Table 1). In addition, nucleotide diversity (π) ranged from 0.00000–0.00376, 0.00000–0.00733, 0.00000–0.00249 and 0.00000–0.00356 in concatenated sequences, *COI*, *ND1* and *ND5*, respectively. The π values were highest for the ZJ population (π = 0.00376) and lowest for the GY, WH, YZ and YC populations (π = 0.00000) based on the concatenated sequences (Table 1). We also analyzed the genetic diversity of the G1, G2, G3, and G4 groups (identified by SAMOVA analysis, see below) based on the concatenated sequences. The H value of G4 was higher than the other three groups (G1, G2, G3), and the π values for G3 and G4 were much higher than G1 and G2 (Supplementary Table S1).

**Table 1:** Parameters of genetic diversity of 23 *Corythucha ciliata* populations based on mtDNA and microsatellite loci.

Population	Code	mtDNA				microsatellite				
		V	n	H	π	AR	A	Ho	H_E_	Fis
Hefei, Anhui	HF	4	2	0.467	0.00086	5.07	5.44	0.47	0.50	0.11
Yongchuan, Chongqing	YCC	5	3	0.511	0.00053	4.76	5.11	0.69	0.71	0.15
Guiyang, Guizhou	GY	0	1	0.000	0.00000	4.29	4.56	0.52	0.57	0.13
Wuhan, Hubei	WH	0	1	0.000	0.00000	4.37	4.67	0.48	0.56	0.13
Hengyang, Hunan	HY	1	2	0.467	0.00022	5.96	6.44	0.62	0.64	0.06
Zhengzhou, Henan	ZZ	1	2	0.200	0.00009	4.29	4.56	0.55	0.68	0.13
Changshu, Jiangsu	CS	18	5	0.822	0.00289	5.29	5.56	0.52	0.74	0.28
Lianyungang, Jiangsu	LY	4	2	0.356	0.00066	5.08	5.44	0.64	0.66	0.04
Nanjing, Jiangsu	NJ	3	2	0.533	0.00074	6.03	6.33	0.63	0.69	0.10
Suqian, Jiangsu	SQ	6	4	0.644	0.00063	6.39	6.89	0.60	0.70	0.17
Taicang, Jiangsu	TC	6	4	0.533	0.00098	4.76	5.11	0.63	0.76	0.15
Xuzhou, Jiangsu	XZ	7	4	0.711	0.00077	6.56	6.89	0.59	0.67	0.13
Yangzhou, Jiangsu	YZ	0	1	0.000	0.00000	5.94	6.22	0.70	0.72	0.10
Zhenjiang, Jiangsu	ZJ	16	6	0.889	0.00376	5.56	6.00	0.60	0.73	0.15
Yancheng, Jiangsu	YC	0	1	0.000	0.00000	4.29	4.56	0.57	0.62	0.13
Ji’ an, Jiangxi	JA	1	2	0.200	0.00009	4.29	4.56	0.57	0.61	0.13
Chengdu, Sichuan	CD	7	6	0.867	0.00140	5.76	6.33	0.57	0.69	0.13
Hanzhong, Shanxi	HZ	7	5	0.844	0.00139	5.63	6.33	0.63	0.74	0.13
Xi’ an, Shanxi	XA	3	4	0.644	0.00059	5.27	5.56	0.64	0.68	0.13
Yiwu, Zhejiang	YW	2	3	0.600	0.00031	5.90	6.22	0.54	0.63	0.05
Taian, Shandong	TA	7	4	0.733	0.00086	4.48	4.67	0.77	0.68	−0.17
Slovenija	SV	5	6	0.889	0.00076	7.06	7.67	0.64	0.75	0.18
Italy	IT	3	4	0.644	0.00050	7.06	7.67	0.65	0.74	0.18

V, variation loci per location; n, the number of haplotype per population; H, haplotype diversity; π, nucleotide diversity; N, the number of individuals per population or group; AR, allelic richness; A, number of alleles; Ho, observed heterozygosity; H_E_ expected heterozygosity; Fis, inbreeding index.

A total of 158 alleles were detected at the nine microsatellite loci, and genetic variability is shown in Supplementary Table S2. All markers were highly informative (PIC > 0.50), which make them useful for analysis of genetic diversity. The number of alleles per locus ranged from 10 (locus CA15) to 23 (locus GA365), and 17.5 was the mean number of alleles per locus. The number of private alleles (Unique Allele Number, UAN) occurred at very low frequencies (<0.025) for all loci. The mean observed and expected heterozygosity values across loci were 0.58 and 0.74, respectively, and the null allele frequency varied from −0.0507–0.1497 (Supplementary Table S2). What’s more, the specific null allele frequencies of nine loci in each population were showed in Supplementary Table S3. In addition, the genetic diversity of *C. ciliata* from the two European populations (Italy and Slovenia) was relatively higher than most of the other populations (*P* = 0.2422 for H; *P* = 0.0019 for A).

A total of 552 individuals representing the 23 populations were genotyped with the nine microsatellite markers (Table 1). The average number of allelic richness (AR) for each population ranged from 4.29 (GY, JA, YC and XZ) to 7.06 (SV and IT). The average number of alleles (A) for each population ranged from 4.56 (WH, ZZ, YC, and JA) to 7.67 (SV and IT). The average observed heterozygosity (H_O_) ranged from 0.47–0.77, mean expected heterozygosity (H_E_) was 0.50–0.75, and the average inbreeding index (Fis) ranged from −0.17 to 0.28. We also analyzed two groups (GI and GII, identified by Structure analysis, please see below), and the genetic diversity of GI was lower than GII (*P* < 0.0001) (Supplementary Table S4). Fisher’s exact test for Hardy-Weinberg equilibrium (HWE) across loci and populations indicated that all 23 populations, and the nine microsatellite loci showed significant deviation from HWE (Supplementary Tables S2 and S3). Besides, no pair of loci showed significant linkage disequilibrium.

### Population genetic structure

With respect to concatenated mtDNA sequences, SAMOVA analysis indicated that the F_CT_ value was highest (0.77243) when K was four; consequently, the 23 populations were divided into four groups designated G1, G2, G3 and G4. G1 included the following populations: HF, YCC, GY, WH, HY, ZZ, CS, TC, YC, JA, CD, HZ, XA, YW, SV and IT. G2 included NJ and YZ, G3 contained LY, SQ, XZ and TA, and G4 consisted of a single population, ZJ. Although G3 was an exception, the occurrence of G1, G2, and G4 was not consistent with geographic distribution.

AMOVA analysis revealed that over 73% of the variation occurred between groups; variation was relatively small within groups and within populations for the concatenated sequences, *COI*, *ND1*, and *ND5* (Table 2). Furthermore, AMOVA revealed that significant genetic structure occurred in *C. ciliata* at various hierarchical levels (among groups, among populations within groups, and within populations) (*P* < 0.05) (Table 2).

**Table 2:** Partitioning of genetic variation at different hierarchical levels.

Group numbers	Gene analyzed	Source of varation	d.f.	Sum of squares	Variance components	Percentage of varation	Fixation indeces
K = 4	Concatenated sequences	Among groups	4	375.915	3.00594 Va	76	F_CT_ = 0.76001**
		Among populations within groups	18	33.12	0.09898 Vb	2.5	F_SC_ = 0.10427**
		Within populations	207	176	0.85024 Vc	21.5	F_ST_ = 0.78503**
	COI	Among groups	4	169.154	1.36290 Va	76.54	F_CT_ = 0.76538**
		Among populations within groups	18	9.228	0.01054 Vb	0.59	F_SC_ = 0.02524*
		Within populations	207	84.3	0.40725 Vc	22.87	F_ST_ = 0.77130**
	ND1	Among groups	4	71.35	0.56829 Va	73.88	F_CT_ = 0.73876**
		Among populations within groups	18	7.528	0.02414 Vb	3.14	F_SC_ = 0.12014**
		Within populations	207	36.6	0.17681 Vc	22.99	F_ST_ = 0.77015**
	ND5	Among groups	4	136.036	1.08167 Va	76.85	F_CT_ = 0.76855**
		Among population within groups	18	16.101	0.06356 Vb	4.52	F_SC_ = 0.19513**
		Within populations	206	54.011	0.26219 Vc	18.63	F_ST_ = 0.81371**
K = 2	Microsatellite	Among groups	1	72.358	0.12226 Va	11.68	F_CT_ = 0.11678**
		Among population within groups	21	104.981	0.08669 Vb	8.28	F_SC_ = 0.09375**
		Within populations	1081	905.854	0.83798 Vc	80.04	F_ST_ = 0.19958**

*Indicated *p* < 0.05; **indicated *p* < 0.01.

**Figure 2:**
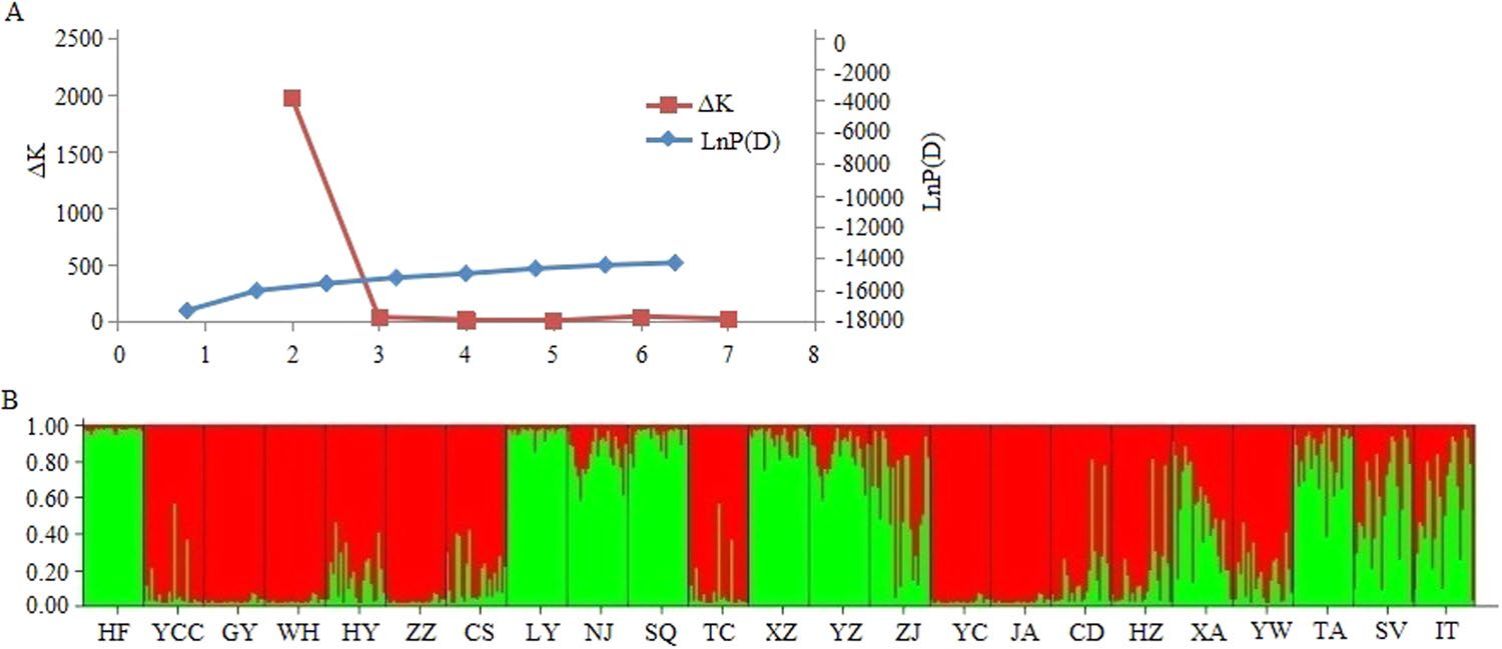
Cluster analysis by STRUCTURE for 23 populations of *C. ciliata*. (**A**) Inference of the number of genetic clusters (K) for *C. ciliata* populations. (**B**) Proportion of genome of each individual assigned to each of the two clusters. Each individual is represented by a vertical bar.

For microsatellite data, we implemented Bayesian cluster analysis with the Structure program. This analysis revealed that the most likely K value was 2 (chosen with Evanno’s ΔK method) (Fig. 2A), which suggested that two main clusters existed among the 23 populations (Fig. 2B). Cluster 1 (GI, shown in red) contained five populations (GY, WH, ZZ, YC and JA), whereas the remaining populations were assigned to cluster 2 (GII, green).

According to the two clusters assigned by Structure, AMOVA suggested that significant genetic structure was present for *C. ciliata* at various hierarchical levels (between groups, among populations within groups, and within populations) (Table 2). A high level of differentiation (80.04%, *F*_ST_ = 0.19958**) was found within populations based on microsatellites, which was different from mtDNA analysis where a high level of differentiation was found between groups.

The genetic differentiation among 23 *C. ciliata* populations was estimated by pairwise F_ST_ values between populations based on concatenated mtDNA sequences and microsatellite data (Supplementary Table S5). F_ST_ values varied from −0.09 to 1.00 for concatenated mtDNA sequences and from −0.02 to 0.32 for microsatellite data. The differentiation between populations was significant (*P* < 0.05 and *P* < 0.01) (Supplementary Table S5). Wright (1938)^29^ assumed that 0.05 < F_ST_ < 0.15 represented a moderate level of genetic differentiation; whereas 0.15 < F_ST_ < 0.25 and F_ST_ > 0.25 indicated a high level of genetic differentiation. Thus, *C. ciliata* groups possess obvious genetic differentiation (Table 3). We also noticed that the values for individual populations are not high for microsatellites but is high for some populations from mtDNA data. Usually, mitochondrial introgression from a close species, male-biased dispersal, demographic expansion or selection on mtDNA were considered as the main reasons for the mito-nuclear (microsatellite) discordance. In the present study, selection may be the cause of the observed discrepancy, and may have differential effects on genetic markers. Loci experiencing balancing selection may have allele frequencies more similar than expected under neutrality, reducing the diversity estimates, whereas selection pressure on mtDNA may accelerate the coalescence of lineages, and thus increase the levels of differentiation.

**Table 3:** The *F*_*ST*_ value between the groups based on concatenated sequences.

Group	G1	G2	G3	G4
G1		0.00000 ± 0.00000	0.00000 ± 0.00000	0.00000 ± 0.00000
G2	0.71887		0.00000 ± 0.00000	0.00000 ± 0.00000
G3	0.87309	0.90427		0.00000 ± 0.00000
G4	0.68488	0.37894	0.59516	

F_ST_ of four groups of *C. ciliata* (below the diagonal); *p* value (±SE) of four groups of *C. ciliata* (above the diagonal).

**Figure 3:**
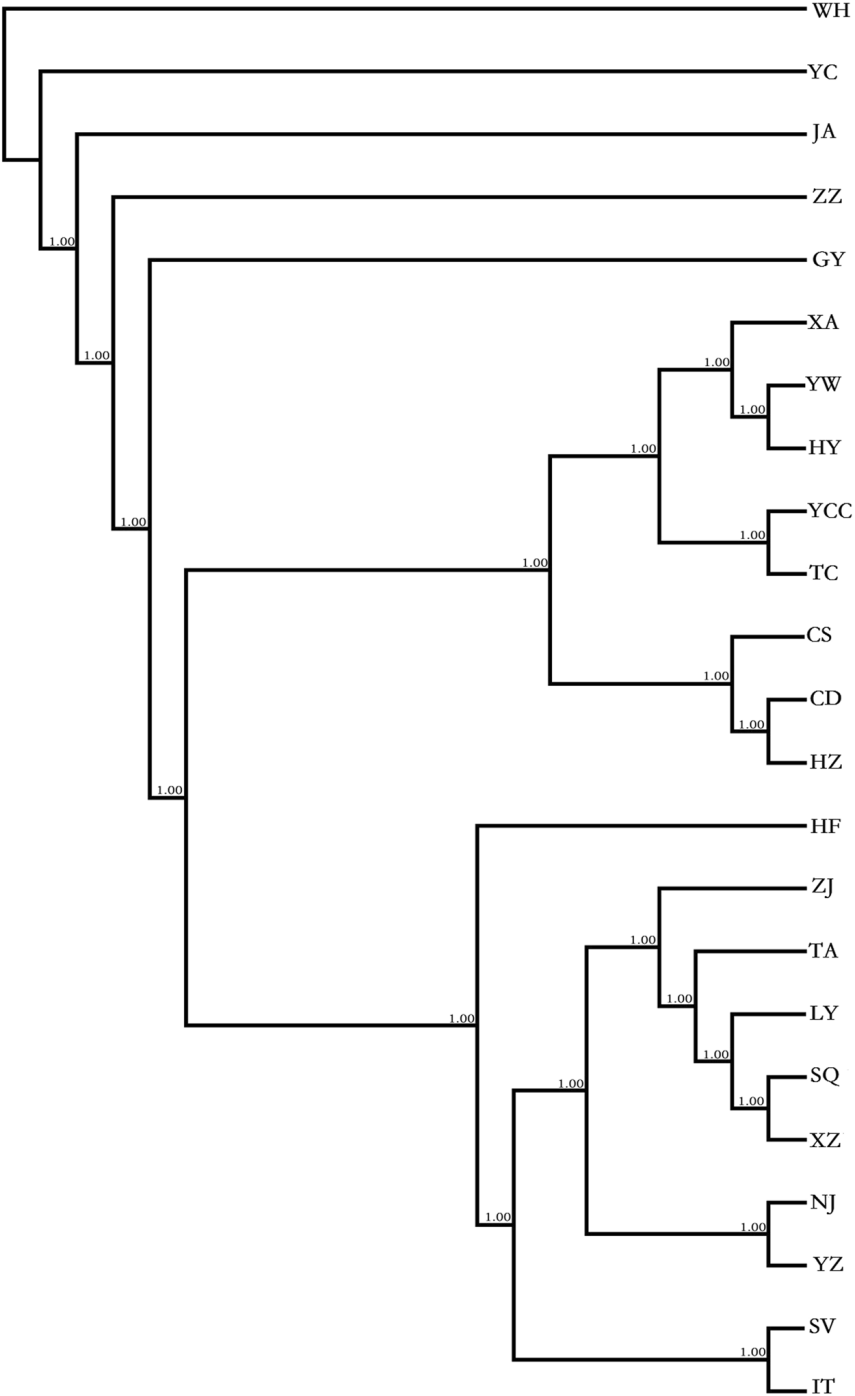
NJ tree inferred from the concatenated mtDNA sequences.

We constructed a NJ tree from concatenated mtDNA sequences using Nei’s genetic distance and PHYLIP v. 3.224 (Fig. 3). In the phylogenetic tree, the clusters among populations were consistent with Nei’s genetic distance. According to the NJ tree, the following populations were assigned to sister groups: (i) WH, YC, JA, ZZ and GY; (ii) YW and HY; (iii) YCC and TC; (iv) CD and HZ; (v) SQ and XZ; (vi) NJ and YZ; and (vii) SV and IT. Some population clusters were consistent with the group assignment (G2 and G3) as defined in SAMOVA; examples include the cluster represented by TA, LY, SQ and XZ and the cluster containing YZ and NJ. It is noteworthy that the European populations SV and IT were closely related to the populations from eastern China represented by ZJ, TA, LY, SQ, XZ, NJ and YZ.

**Figure 4:**
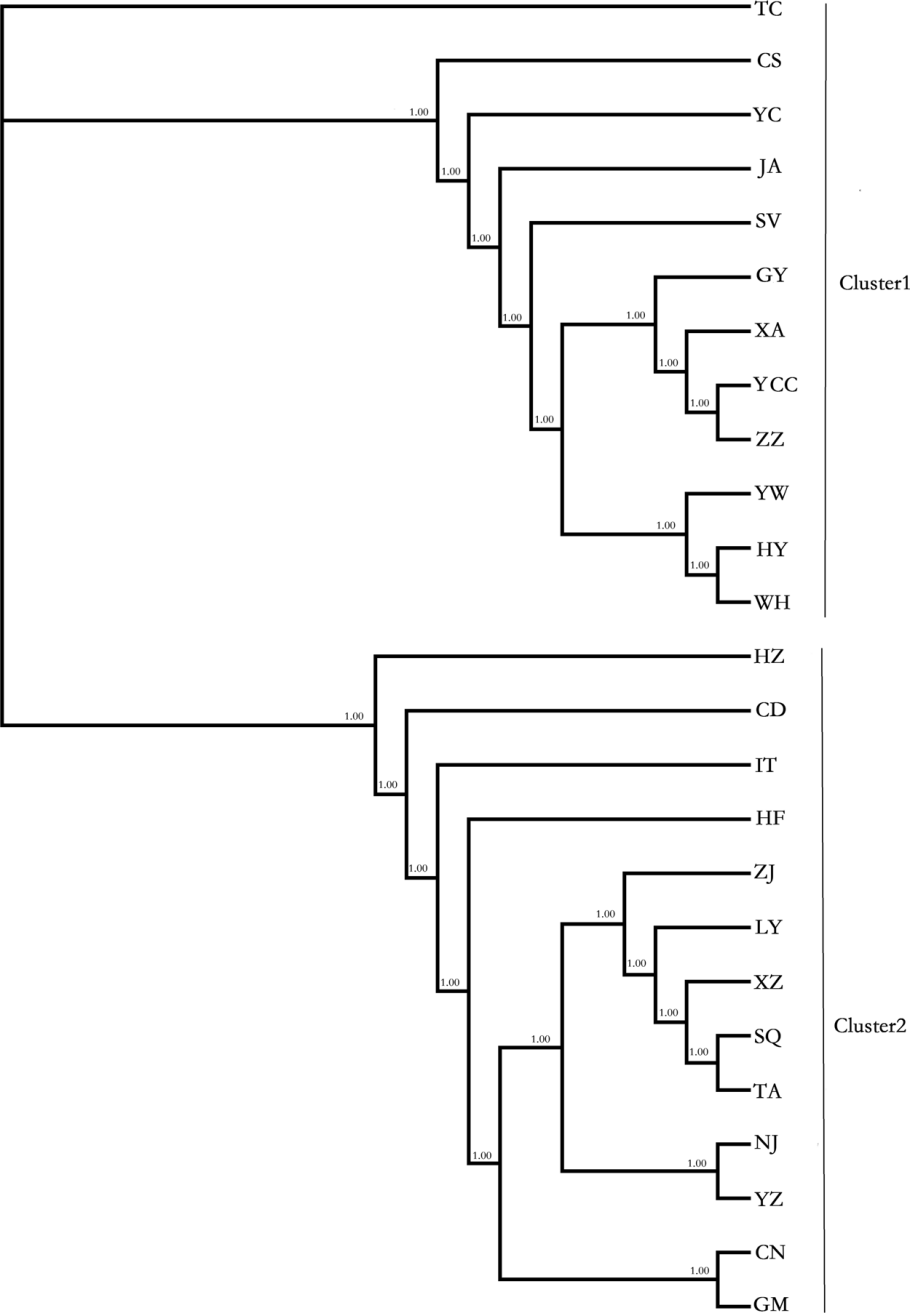
NJ tree inferred from the *COI* sequences.

Additionally, we constructed a NJ tree for *COI* sequences of 25 populations. This included the 23 populations listed in Table 1 and two additional populations from Canada (CN) and Germany (GM), which were obtained from GenBank (Fig. 4). In the phylogenetic tree, the clusters among populations were consistent with Nei’s genetic distance. According to the NJ tree, we found the same population clusters (NJ and YZ; ZJ, TA, LY, SQ, XZ) with the defined groups of the concatenated mtDNA sequences. The SV and IT populations were not sister groups, whereas the CN and GM populations were sisters. In the NJ tree, we also observed two clusters. In cluster 1, the SV population grouped with 11 Chinese populations. In cluster 2, the IT, GM and CN populations grouped with the remaining ten Chinese populations. The genetic diversity of cluster 2 was higher than cluster 1 although it is not significant (*P* = 0.1124 for H and *P* = 0.1831 for π).

**Figure 5:**
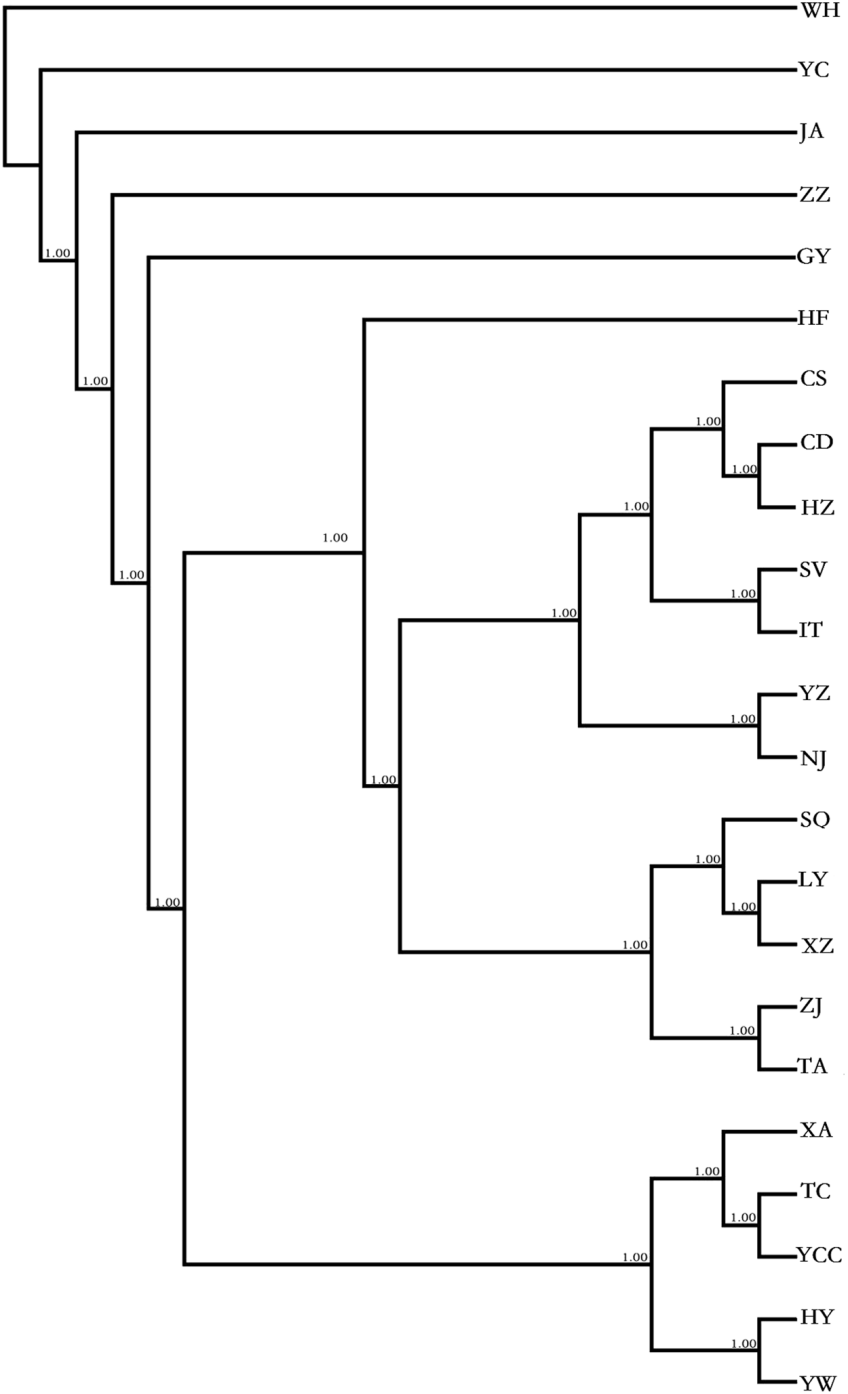
NJ tree inferred from multilocus microsatellites.

We also constructed a NJ tree from microsatellite data (Fig. 5). Analysis indicated the presence of two primary clusters among the 23 populations, and the distribution was consistent with Nei’s genetic distance. When we compared the NJ tree derived from mtDNA sequences with the dendogram obtained for microsatellites, the cluster formed by the GY, WH, ZZ, YC and JA populations was conserved. The sister groups (SV and IT; NJ and YZ; CD and HZ; YW and HY; and YCC and TC) were also preserved (Figs 3 and 4).

### Gene flow and isolation by distance

The effective mutation scaled population size (Θ) was estimated for each population and group based on concatenated mtDNA sequences and microsatellite data. The bidirectional mutation-scaled immigration rate (M) was estimated for 253 population pairs (Supplementary Tables 6 and 7). The M ranged from 282.0 (ZZ → ZJ) to 777.3 (CS → GY), which indicated high gene flow (Θ * M) between population pairs with concatenated mtDNA sequences. Furthermore, 22 population pairs (8.7%) showed asymmetric migration rates (Supplementary Table S6).

With respect to the four groups identified by mtDNA analysis, M varied from 226.2 (G1 → G3) to 742.2 (G4 → G2) (Table 4). There were no asymmetric migration rates between group pairs. More importantly, the migrants from the eastern regions (including G2, G3 and G4) to the western regions (G1) were high (Table 4).

**Table 4:** Estimates of gene flow between four *Corythucha ciliata* groups based on concatenated mtDNA sequences.

Group, i	Θ	Group1 G1 → i	Group 2 G2 → i	Group 3 G3 → i	Group 4 G4 → i
G1	0.00226 (0.00033–0.00412)		444.9 (0.0–898.7)	475.7 (0.0–922.0)	471.5 (0.0–876.7)
G2	0.00086 (0.00000–0.00253)	421.3 (53.3–855.3)		467.7 (0.0–861.3)	742.2 (285.3–1000.0)
G3	0.00041 (0.00000–0.00207)	226.2 (0.0–578.7)	325.3 (0.0–808.7)		739.4 (329.3–1000.0)
G4	0.02890 (0.00000–0.07973)	316.1 (0.0–750.7)	427.6 (0.0–852.0)	441.9 (0.0–852.7)	

Θ: mutation scaled effective population size; M: mutation scaled effective immigration rate. In parentheses the 95% HPD intervals. The source population is indicated in columns, the target population in row.

Additionally, we also observed higher migration rates (M) and asymmetric migration between population pairs based on microsatellites. The M ranged from 131.93 (GY → CS) to 888.44 (HF → YW), and 48 population pairs (18.9%) had asymmetric migration rates (Supplementary Table 7). The F_ST_ value between GI and GII was 0.12927 (*P* < 0.0001).

The Mantel test indicated that there was no correlation between genetic and geographic distance (Km) for concatenated mtDNA sequences (r = −0.0683, *P* = 0.6943), *COI* (r = −0.1139, *P* = 0.8038), *ND1* (r = −0.1378, *P* = 0.8723) and *ND5* (r = −0.1547, *P* = 0.8820) and microsatellites (r = −0.1247, *P* = 0.8821).

### Tests of alternative hypotheses on the source and dispersal of *C. ciliata*

**Figure 6:**
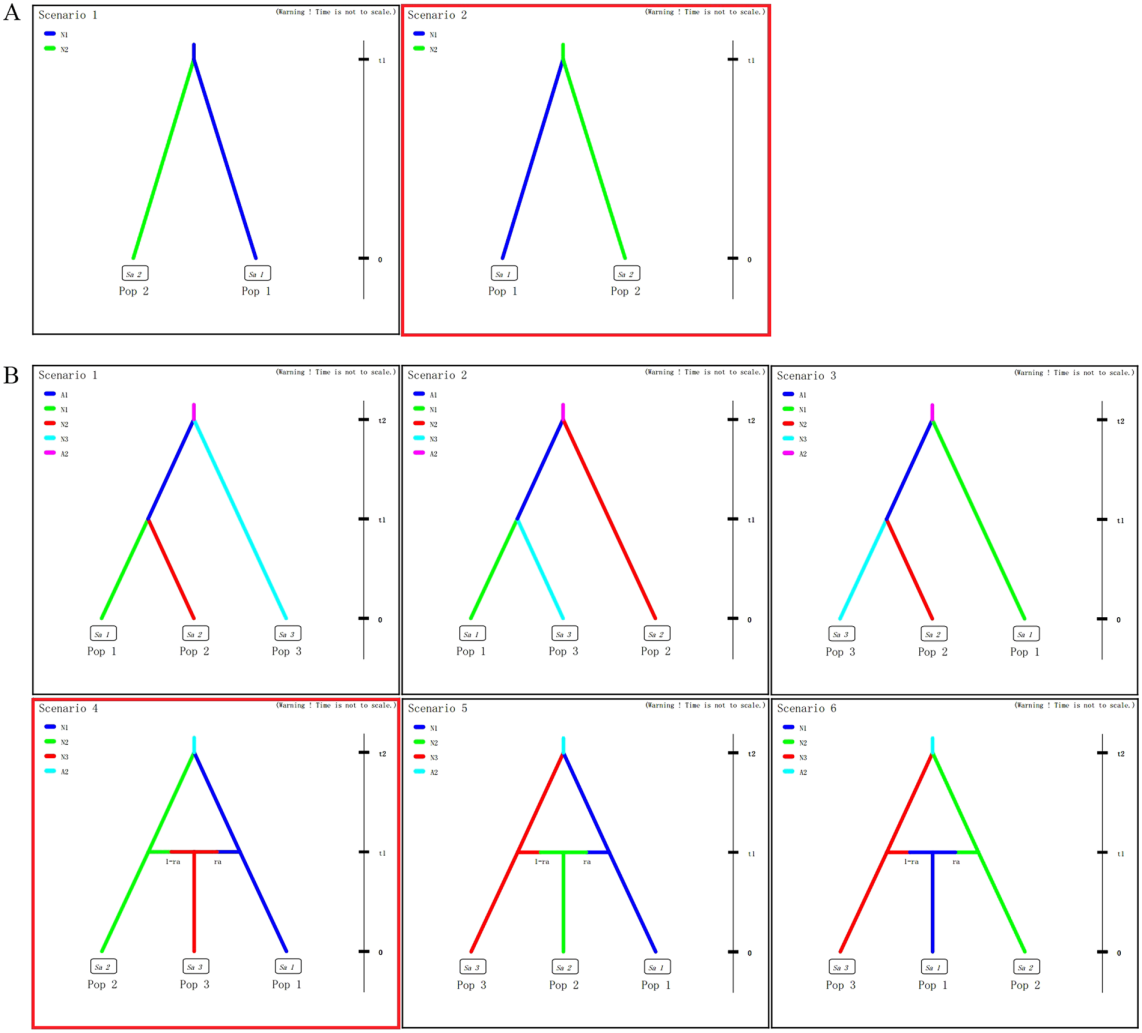
Scenarios for the DIY ABC analyses, which was designed to infer the origin and dispersal of *C. ciliata*. (**A**) Two scenarios showing the origin of *C. ciliata*. (**B**) Six scenarios showing relationships of three groups (G2, G3 and G4); regarding the variation in population size and the split and admixture events. The best scenario in the corresponding step was indicated by red square.

To decipher the invasion and colonization routes of *C. ciliata*, we used the Approximate Bayesian Computation (ABC) analysis^30, 31^. The first step of ABC analysis was undertaken to define the most likely area for the initial invasion by *C. ciliata*. These results unambiguously pointed to a scenario where invasion initiated in the eastern coastal region (G2, G3 and G4) (logistic regression: 0.5192 [95% CI: 0.4556–0.5827]) (Scenario 2; Fig. 6A and Table 5). The second step of ABC analysis was intended to assess genetic relationships of the three most likely sources of introduction (G2, G3 and G4). Scenario testing analyses of the relationships among the three eastern groups suggested that G4 developed from an admixture of G2 and G3 (logistic regression: 0.6023 [95% CI: 0.5359–0.6688]) (Scenario 4; Fig. 6B and Table 5).

**Table 5:** Description of the scenarios used in the approximate Bayesian.

Step	Scenario	Posterior probability	95% CI (lower-upper)
Step1	1	0.4808	(0.4173, 0.5444)
	2	0.5192	(0.4556, 0.5827)
Step2	1	0.0242	(0.0125, 0.0359)
	2	0.0608	(0.0404, 0.0811)
	3	0.1436	(0.0962, 0.1909)
	4	0.6023	(0.5359, 0.6688)
	5	0.1121	(0.0771, 0.1471)
	6	0.0571	(0.0389, 0.0752)

Computation analysis in DIYABC to test the source and the differentiation time among species. The relative posterior probabilities and 95% confidence intervals for each scenario were computed via the logistic regression on 1% of the closest data sets to the observed data.

### Ecological niche models

**Figure 7:**
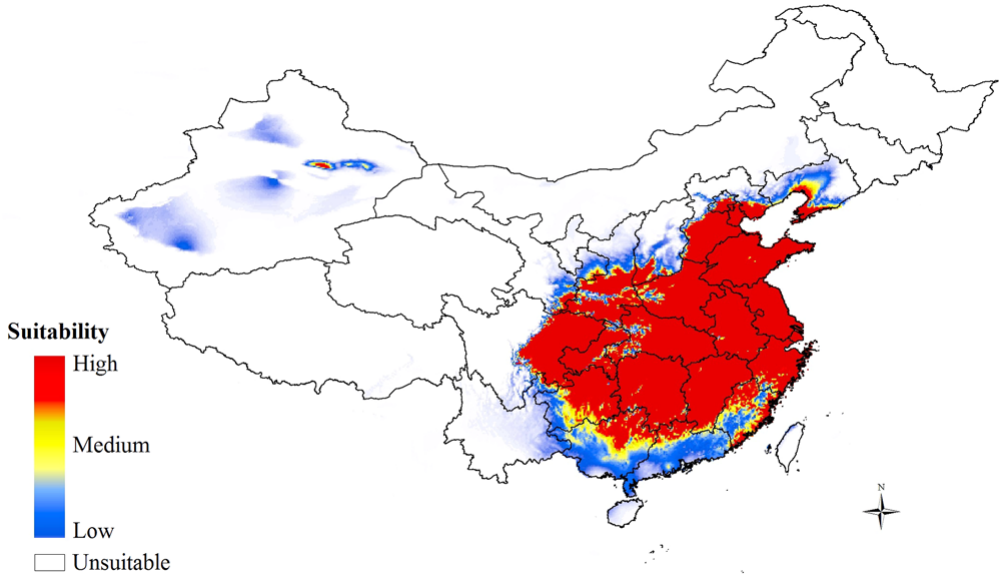
Potential distribution of *C. ciliata* under current climate conditions. The map is made by ArcGIS 10.2 software, http://www.arcgis.com/features/.

To evaluate the abiotic environmental factors that have influenced the distribution of *C. ciliata*, we implemented the MaxEnt model, which enables probability distributions to be estimated from incomplete information^32^. The area under the curve (AUC) for the test data was 0.994, indicating a high fit of the modelled and observed distributions^32, 33^. As evident from Fig. 7, most regions of eastern, central, and southern China were predicted to be highly suitable for *C. ciliata*. The Jackknife evaluation indicated that precipitation during the warmest quarter (Bioclim 18) and temperature seasonality (Bioclim 4) were the main factors influencing the distribution of *C. ciliata* (32.9% and 22.8%, respectively).

### MJ networks of haplotypes and demographic history

**Figure 8:**
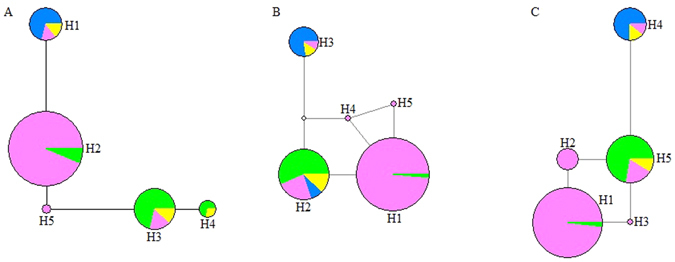
Median-joining networks of haplotypes. Sections of pies are proportional to haplotype frequency. (**A**,**B** and **C**) represent *COI*, *ND1* and *ND5*, respectively. Pink, Group 1; blue, group 2; green, group 3; and yellow, group 4.

MJ networks were reconstructed from haplotypes of *COI*, *ND1*, and *ND5* (Fig. 8). Some haplotypes occurred at a higher frequency (G1 and G3) and were centrally located in networks (e.g. H2 for *COI*, H1 for *ND1* and H1 for *ND5*).

**Figure 9:**
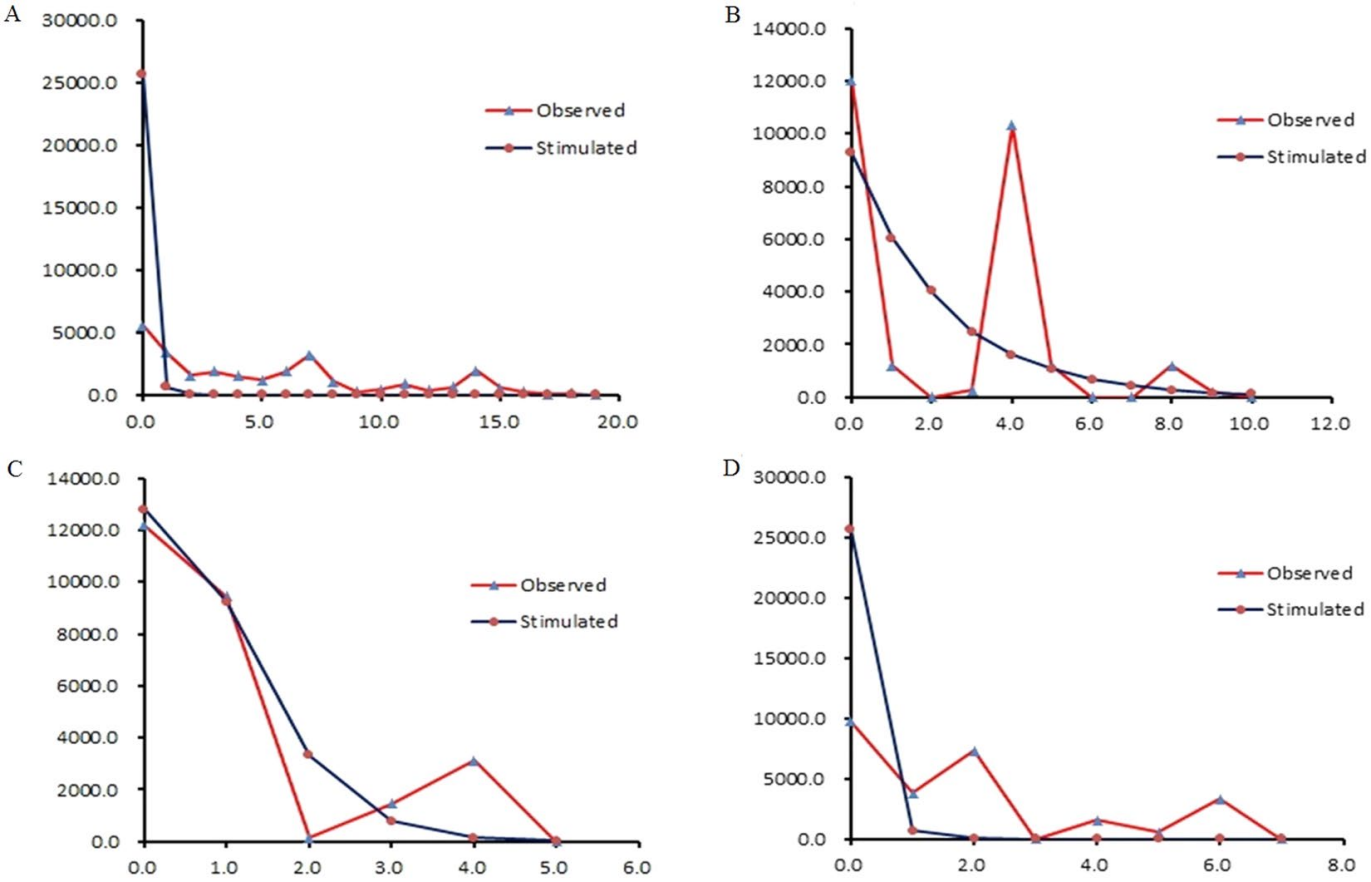
Observed and simulated mismatch distributions. (**A**,**B**,**C** and **D**) represent concatenated sequences, *COI*, *ND1* and *ND5*, respectively. The horizontal axis represents the number of pairwise differences, and the vertical axis represents the relative frequency.

Most of Tajima’s *D* and Fu’s *Fs* values of concatenated sequences, *COI*, *ND1* and *ND5* were positive and not significant. However, exceptions included the *Fs* value for G1 in the concatenated sequences, and Tajima’s D values for G1 and G3 in the concatenated mtDNA sequences, *COI*, *ND1* and *ND5*, respectively (Table 6). Thus, populations of *C. ciliata* in China experienced a population reduction or bottleneck effect. The mismatch distribution for concatenated sequences, *COI*, *ND1*, and *ND5* was not unimodal (Fig. 9), which suggests that *C. ciliata* populations in China did not experience a recent expansion.

**Table 6:** Parameters of demographic history of the collated 23 populations and each of four population’s groups independently based on concatenated mtDNA sequences.

Gene	Group	θ_0_	θ_1_	τ	D	*Fs*	SSD
Conc. Sequences	All	0.000	99999.000	0.000	1.61467	−2.43275	0.6823**
	G1	0.000	99999.000	0.000	−1.21146	8.53373**	0.4543**
	G2	1.800	3.600	4.475	0.52089	2.97317	0.2973
	G3	0.000	99999.000	0.000	−0.50357	2.41273	0.4677**
	G4	0.000	14.918	15.730	2.04604	1.58983	0.0520
COI	All	1.225	1.797	80.000	1.19105	5.30504	0.1849*
	G1	0.000	0.190	3.000	−1.23768	0.51415	0.0252
	G2	—	—	—	0.00000	0.00000	—
	G3	0.000	1493.750	0.000	−0.11624	2.22848	0.2486**
	G4	0.004	8.129	9.701	1.98315	4.57309	0.1687
ND1	All	0.000	99999.000	0.691	0.90050	1.16351	0.0285**
	G1	0.000	0.324	3.000	−0.91538	−2.11362	0.0032
	G2	1.800	3.600	4.475	0.52089	2.97317	0.2973
	G3	0.000	0.385	0.213	−0.56352	−0.21825	0.0001
	G4	0.002	1.373	4.188	1.15198	2.98462	0.2393
ND5	All	0.000	99999.000	0.000	1.61123	3.73185	0.5138**
	G1	0.000	6.002	0.512	−0.63642	−0.05680	0.0130
	G2	—	—	—	0.00000	0.00000	—
	G3	0.000	0.104	3.000	−0.74525	0.77336	0.0276
	G4	0.002	2.908	5.172	1.95339	4.19230	0.3121*

θ_0_, Effective population size before expansion; θ_1_, effective population size after expansion; τ, population expansion time; D, Tajiama’s D; *Fs*, Fu’s; SSD, sum of squared deviations between observed and expected mismatch under a sudden expansion model; *indicated *p* < 0.05, **indicated *p* < 0.01.

### Bottleneck effects based on microsatellite data

We used three microsatellite mutation models (IAM, SMM and TPM) to analyze bottleneck effects in *C. ciliata*. In the three mutation models, we used the Wilcoxon test to analyze whether the heterozygosity excess was significant. Two populations (CS and TA) and one population (XZ) had a statistically significant excess of heterozygotes when analyzed using the infinite allele model (IAM) and two-phased model of mutation (TPM), respectively (Table 7). However, heterozygosity excess was observed in 12 populations (HF, GY, WH, ZZ, CS, LY, NJ, SQ, XZ, YZ, YC and JA) using the step-wise mutation model (SMM, Table 7), suggesting that most *C. ciliata* populations in this study underwent a genetic bottleneck.

**Table 7:** Bottleneck test for *Corythucha ciliata* based on microsatellites.

Population	IAM		TPM		SMM	
	Hde/Hex	Wilcoxon signle-rank test	Hde/Hex	Wilcoxon signle-rank test	Hde/Hex	Wilcoxon signle-rank test
HF	2/7	0.18763	4/5	0.53545	8/1	**0.00413**
YCC	2/7	0.17249	2/7	0.20530	4/5	0.51797
GY	2/7	0.18510	5/4	0.29402	8/1	**0.00417**
WH	2/7	0.17400	5/4	0.29692	8/1	**0.00432**
HY	1/8	0.05825	4/5	0.53739	4/5	0.52270
ZZ	2/7	0.18673	5/4	0.29573	8/1	**0.00402**
CS	0/9	**0.00775**	4/5	0.54209	7/2	**0.02636**
LY	2/7	0.18909	3/6	0.46370	7/2	**0.02641**
NJ	3/6	0.45426	6/3	0.10612	7/2	**0.02710**
SQ	2/7	0.21535	5/4	0.27772	8/1	**0.00417**
TC	2/7	0.17064	2/7	0.21160	4/5	0.53824
XZ	6/3	0.10984	8/1	**0.00446**	8/1	**0.00384**
YZ	3/6	0.44926	6/3	0.10545	7/2	**0.02984**
ZJ	3/6	0.44213	4/5	0.53767	5/4	0.26376
YC	2/7	0.18610	5/4	0.29428	8/1	**0.00402**
JA	2/7	0.18557	5/4	0.30080	8/1	**0.00366**
CD	2/7	0.21265	5/4	0.28253	6/3	0.11454
HZ	2/7	0.21071	5/4	0.26882	6/3	0.10957
XA	3/6	0.43292	4/5	0.52410	6/3	0.09942
YW	1/8	0.05424	4/5	0.53960	4/5	0.52825
TA	0/9	**0.00472**	1/8	0.05540	2/7	0.20039
SV	2/7	0.22796	5/4	0.26981	6/3	0.11534
IT	2/7	0.23507	5/4	0.27117	6/3	0.10713

Hde, heterozygote deficiency; Hex, heterozygote excess. Bold indicates *p* < 0.05.

## Discussion

### Genetic diversity and population differentiation

Based on analysis of variation in three mtDNA sequences and nine microsatellites, we conclude that the genetic diversity of *C. ciliata* populations in China is relatively low (mean values of H, π and A are 0.447, 0.00080 and 5.59, respectively) and generally less than the diversity exhibited in European populations (*P* = 0.2422 for H; *P* = 0.0019 for A) (Table 1 and Supplementary Table S1). Invasiveness of a species is often associated with a loss in genetic diversity, which may explain these findings^34–37^. Interestingly, the ZJ population (group 4) exhibited a high level of genetic diversity (H = 0.889 and π = 0.00376) and a high level of gene flow with the other three groups (9.135 to 21.450). The increased genetic variability due to the admixing between G2 and G3 (Fig. 6), along with multiple introductions, may explain the relatively high genetic variability of insects in G4. It is important to mention that ZJ (Zhenjiang) is one of the most important hub cities in eastern China; thus it remains a convenient location for dispersal of *C. ciliata* via humans and vehicles.

The HWE test revealed that the 23 populations significantly deviated from HWE. We analyzed three possible reasons for this deviation by excluding the presence of null alleles. Significant deviation from HWE could be caused by inbreeding (Fis: −0.17–0.28), and sampling bias and overlapping generations could result in the inbreeding phenomenon. In support of this, we observed that *C. ciliata* likes to congregate at feeding sites and exhibits generational overlap after the second generation^38^. Besides, we could not rule out possibility that homozygote excess was related to HWE because most of the loci had a tendency to slight homozygote excess (Table 1).

### Genetic structure and dispersal of *C. ciliata* in China

We discovered that *C. ciliata* exhibits obvious genetic structure in China based on Structure analysis. SAMOVA indicated that the 23 populations were defined as four groups based on mtDNA sequences, and AMOVA indicated that significant genetic differentiation existed among groups, among populations within groups, and within populations. Additionally, the NJ tree revealed a pattern of genetic differentiation similar to the SAMOVA analysis. Conversely, Structure analysis indicated that the 23 populations formed two clusters based on microsatellites; AMOVA results showed significant genetic differentiation among groups, among populations within groups, and within populations. The NJ tree based on microsatellites revealed genetic differentiation similar to Structure analysis. The pairwise F_ST_ values also indicated that the Chinese populations of *C. ciliata* have obvious genetic differentiation. Moreover, genetic differentiation was not caused by geographic isolation because genetic and geographic distances were not correlated.

The direction of asymmetric gene flow can potentially reveal migration patterns and routes of invasion. Our analysis of gene flow indicated that *C. ciliata* migrated from the eastern regions (e.g. G2, G3 and G4) to the western regions of China (G1) (Table 4). Furthermore, ABC analyses indicated that the most likely scenario regarding the invasion of *C. ciliata* was a complex situation involving admixture and split. More specifically, G4 (ZJ) was an admixture derived from G2 and G3, which was then introduced to inland China (G3). This route of invasion is supported by the fact that sycamore trees were transplanted to China. Thus, we hypothesize that *C. ciliata* invaded China from seaports located along the east coast, possibly due to the importation of sycamore trees. However, it is important to note that our hypotheses are somewhat inconsistent with the initial reports of *C*. *ciliata* in Changsha^15^ and Wuhan^16, 17^, which are inland cities.

Some populations located in eastern China (e.g. ZJ) exhibited a high level of genetic diversity. These populations exhibit an asymmetric migration relative to other populations and have a slighter higher genetic diversity in mtDNA than other populations (Mean value of H: 0.517 > 0.414, *P* = 0.2411). This is likely to be another example of the Bridgehead effect^39^, which was predicted to be a common occurrence^40^ and formed the bridgehead populations that act as a source of western populations in China. The Bridgehead effect which refers to widespread secondary invasions stemming from a particular primary invasive population has been observed for the dispersal patterns of several invasive insects, including *Frankliniella occidentalis* ^41^ the fire ant *Solenopsis invicta* ^42^ and the western corn rootworm, *Diabrotica virgifera virgifera* ^43^.

In summary, these analyses provide compelling evidence that the invasion of *C. ciliata* started in eastern China and likely spread inland from eastern coastal areas.

### Demographic history of *C. ciliata*

The analysis of Tajima’s *D* and Fu’s *Fs* values and mismatch distributions indicated that Chinese populations of *C. ciliata* fit the neutral evolution model and have not undergone a recent population expansion. Most of the *D* and *Fs* values were positive, which suggests that *C. ciliata* underwent a population reduction and wide migration or a recent bottleneck effect. This hypothesis is supported by several findings reported in this paper. For example, the overall genetic diversity for Chinese populations of *C. ciliata* was not high based on analysis of mtDNA genes and microsatellite data. Additionally, analysis of gene flow showed that the average migration rate between populations was high. Finally, the microsatellite data indicated that most populations experienced a bottleneck effect.

### Invasiveness of *C. ciliata* and implications for management

It is important to ask why *C. ciliata* can successfully invade a new location despite low genetic diversity and wide migration or a recent bottleneck effect. Our study provides clues to address these questions from the standpoint of population genetics and environmental factors. First, Chinese populations of *C. ciliata* have population structure at different levels. Additionally, gene flow is higher between some pairs of populations and groups based on mitochondrial genes. This could be attributed to multiple introductions of *C. ciliata* in eastern coastal areas of China and subsequent dissemination. Repeated introductions or colonization of a given area from multiple sources can negate the decrease in genetic variability associated with colonization or rescue a species from an actual loss in genetic diversity^37^. ABC analyses indicated that the *C. ciliata* invasion started with the G2 and G3 groups independently and then evolved into G4. With respect to environmental factors and dissemination, it is noteworthy that *C. ciliata* can colonize and invade many different environments (Fig. 7).

In conclusion, the analyses of genetic diversity and population structure for *C. ciliata* conducted in this study provide a better understanding of the adaptability of this important pest. *C. ciliata* is adept at successfully invading new locations, which may be helpful in formulating more effective control measures for this invasive species.

## Materials and Methods

### Sampled populations and DNA extractions

In this study, *C. ciliata* adults (females and males) were collected from leaves of *Platanus* spp. in 23 urban locations, including Italy and Slovenia. The 21 Chinese populations (Fig. 1 and Table 8) encompassed most of the regions infested with *C. ciliata*. Adult samples of *C. ciliata* were preserved in 100% ethanol and stored at −20 °C. Genomic DNA was extracted from individual samples using DNAVzol (Bioteke, Beijing, China) and stored at −20 °C until needed for PCR.

**Table 8:** The information of sampling populations.

Locating, Province	Code	Coordinates	N1	N2	Collecting year
Hefei, Anhui	HF	N 31°48′ E 117°15′	24	10	2009
Nanjing, Jiangsu	NJ	N 32°03′ E 118°47′	24	10	2009
Yangzhou, Jiangsu	YZ	N 32°23′ E 119°24′	24	10	2009
Zhenjiang, Jiangsu	ZJ	N 32°11′ E 119°25′	24	10	2009
Changshuo, Jiangsu	CS	N 31°39′ E 120°45′	24	10	2009
Taichang, Jiangsu	TC	N 31°27′ E 121°07′	24	10	2009
Yancheng, Jiangsu	YC	N 33°20′ E 120°09′	24	10	2009
Xuzhou, Jiangsu	XZ	N 34°12′ E 117°17′	24	10	2009
Lianyungang, Jiangsu	LY	N 34°35′ E 119°13′	24	10	2009
Suqian, Jiangsu	SQ	N 33°57′ E 118°16′	24	10	2009
Taian, Shandong	TA	N 36°12′ E 117°05′	24	10	2010
Jian, Jiangxi	JA	N 27°06′ E 114°59′	24	10	2009
Zhenzhou, Henan	ZZ	N 34°44′ E 113°37′	24	10	2010
Yiwu, Zhejiang	YW	N 29°18′ E 120°04′	24	10	2009
Guiyang, Guizhou	GY	N 26°38′ E 106°37′	24	10	2009
Chengduo, Sichuan	CD	N 30°34′ E 104°03′	24	10	2010
Yongchuan, Chongqing	YCC	N 29°21′ E 105°55′	24	10	2010
Hanzhong, Shanxi	HZ	N 33°04′ E 107°01′	24	10	2010
Xian, Shanxi	XA	N 34°20′ E 108°56′	24	10	2010
Wuhan, Hubei	WH	N 30°35′ E 114°18′	24	10	2010
Hengyang, Hunan	HY	N 26°53′ E 112°34′	24	10	2009
Slovenija	SV	N 14°31′ E 46°03′	24	10	2010
Italy	IT	N 7°68′ E45°07′	24	10	2014

N1, the number of individuals genotyped at nine microsatellites; N2, the number of partial MtDNA genes (*COI*, *ND1* and *ND5*) sequences used.

### Amplification of mtDNA genes and sequencing

Three fragments of the mitochondrial genome (631, 751 and 786 bp of *COI*, *ND1* and *ND5*, respectively) were amplified using DNA from 10 individuals per population. Primer pairs included COI-F (5′GGTCAACAAATCATAAAGATATTGG) and COI-R (5′-TAAACTTCAGGGTGACCAAAAAATCA)^44^, ND1-F (5′-ATTCAGACTCTCCTTCAGCA) and ND1-R (5′-GGATAACAGCCTAATCTTCT), and ND5-F (5′-AAAATCACCTCAACTATCAT) and ND5-R (5′-GCTCCTACACCAGTTTCTTC). Primers for *ND1* and *ND5* were designed based on the complete mitochondrial genome sequence of *C. ciliata* ^45^. Conditions for PCR amplification were as follows: initial denaturation for 5 min at 95 °C, followed by 35 cycles at 94 °C (1 min each), annealing for 1 min at 45–50 °C, elongation for 1–3 min (depending on predicted fragment length) at 68 °C, and a final extension step at 72 °C for 10 min. Amplification products were purified and then sequenced by Invitrogen Biotechnology Co. (Shanghai, China).

### Amplification of microsatellites and determination of fragment size

The nine microsatellite loci, primer sequences, and PCR conditions used in the current study were previously described by Yang *et al.* ^46^. Amplifications were conducted using DNA from 24 individuals per population (total number = 552). Amplified PCR products were analyzed by Sangon Biotechnology Co. (Shanghai, China) using the ABI 3730 automated DNA sequencer (Applied Biosystems). GENEMAPPER v. 3.5 (Applied Biosystems) was used to obtain allele designations.

### Analysis of diversity and population structure

Fragments comprising the three mitochondrial genes were initially aligned using ClustalX v. 2.0^47^. The number of variable sites and haplotypes, haplotype diversity, and nucleotide diversity of the three mtDNA genes (*COI*, *ND1* and *ND5*) were calculated by DnaSP v. 5.0^48^. The expected and observed heterozygosity, polymorphic information content, and estimated null frequency were calculated for each locus in individual populations using CERVUS v. 3.0.3^49^.

The number of alleles (A), allelic richness (AR), inbreeding index (Fis), and observed and expected heterozygosity (Ho and H_E_) were calculated by FSTAT v. 2.9.3.2^50^. The deviation from Hardy-Weinberg equilibrium for each population and linkage disequilibrium for pairs of loci was calculated with GENEPOP v. 3.4^51^, and *P* values were adjusted using the Sequential Bonferroni procedure^52^. Null alleles were identified using Micro-Checker v. 2.2.3^53^. T-tests were used to evaluate the significance of differences in diversity estimates haplotype diversity (H), nucleotide diversity (π) and number of alleles (A) of the populations using JMP v.12.

Population groups were identified using concatenated sequences of the three mtDNA genes; information on longitude and latitude were included, and the data were analyzed using SAMOVA v. 1.0^54^. The most likely number of clusters (K) was determined by repeating the analysis with K ranging from 2 to 6 and selecting the subdivision scheme associated with the highest F_CT_. The clustering of individuals from all populations was determined using Structure v. 2.0^55^. The most supported number of clusters (K) was obtained based on multilocus microsatellite data. We used an admixture ancestry model and the correlated allele frequency model with a burn-in period of 100,000 iterations and 1,000,000 Markov chain Monte Carlo (MCMC) repetitions to calculate the most likely number of genetic clusters. We performed 10 independent runs for each K to confirm consistency across runs, tested K from 1 to 8, and used the ΔK method of Evanno *et al*.^56^ to choose the most supported value of K.

Analysis of molecular variance (AMOVA) was implemented by ARLEQUIN v. 3.0^57^. The definition of groups in AMOVA was determined by SAMOVA and Structure analysis for mtDNA sequences and microsatellite data, respectively. Pairwise F_ST_ (differentiation index) was analyzed by ARLEQUIN v. 3.0^57^.

The correlation between genetic [F_ST/_(1 − F_ST_)] and geographic distance matrices in log scale^58^ was performed using the Isolation by Distance Web Service (IBDWS)^59^ with 10,000 randomizations.

### Gene flow analysis and median joining networks of haplotypes

The gene flow between pairs of populations and groups was estimated by MIGRATE v. 3.2.1^60^. We used a Bayesian search strategy with the following parameters: three long chains (1,000,000 trees) with 10,000 trees discarded in the initial “burn-in”; replicates = YES, 5; randomtree = YES; and heating = ADAPTIVE: 1 {1.0 1.2 1.5 3.0}. The MIGRATE program was conducted for four cycles, and the seed number was changed for each run.

Median-joining (MJ) networks of haplotypes for the three mtDNA genes were constructed using Network v. 4.6^61, 62^.

### Scenarios of ABC analyses

To explore models that best describe the demographic history and geographic distribution of present-day populations, we evaluated the posterior probabilities of historical scenarios using the Approximate Bayesian Computation approach (ABC)^30^ as implemented in DIY ABC v. 2.1.0^31^. Gene flow analyses revealed that migrants moved from the eastern regions (including G2, G3 and G4) to western China (G1). Therefore, to simplify scenarios for ABC analysis, we first combined the three groups from eastern regions and then tested two alternative scenarios for potential sources in China (Fig. 6A). A total of six scenarios were considered with respect to population size, split, and admixture events for *C. ciliata* in eastern inland regions (Fig. 6B).

### Demographic analysis and bottleneck effects

The mismatch distribution in ARLEQUIN v. 3.0 was used for demographic analysis, and Tajima’s *D* and Fu’s *Fs* tests were implemented to analyze neutrality. Effective population size before expansion (Θ_0_), effective population size after expansion (Θ_1_), population expansion time (τ), and the sum of squared deviation (SSD) between observed and expected mismatches were also calculated. The significance of these statistics was tested by parametric bootstrapping (1,000 replicates) in ARLEQUIN v. 3.0.

Bottleneck effects were detected using Bottleneck v. 3.4^63^. Analyses were based on the frequency of each allele with the following three models: the infinite allele model (IAM)^64^; the step-wise mutation model (SMM)^65^, and the two-phased model of mutation (TPM)^66^. The number of iterations for Bottleneck was 1,000, and the Wilcoxon sign-rank test was implemented.

In all instances where multiple tests were conducted simultaneously, the sequential Bonferroni procedure^52^ was applied to adjust the nominal significance level.

### Phylogenetic reconstruction

Pairwise F_ST_ values for populations were calculated using ARLEQUIN v. 3.0 with 1,000 permutations. NJ phylogenetic trees were generated using PHYLIP v. 3.2^67^ and based on Nei’s genetic distance. The NJ tree was constructed using *COI* sequences from the 23 populations listed in Table 1 and two additional populations from Germany (GM) and Canada (CN); the latter sequences were obtained from GenBank.

### MaxEnt modeling algorithm

To estimate the environmental suitability of areas for *C. ciliata* invasion, we built a species distribution model (SDM) based on field observations of *C. ciliata* occurrences coupled with environmental maps. Data from sites known to be infested with *C. ciliata* in China were entered into MaxEnt^32^. Nineteen bioclimatic data layers were acquired from the WorldClim dataset (http://www.worldclim.org/)^68^ at a spatial resolution of 2.5 arc minutes. The random test percentage was set to 25%, and the Jackknife procedure was used to estimate the contribution of each variable based on performance of the model. The area under the curve (AUC) value was calculated for model validation; AUC reflects the model’s ability to distinguish between present records and random background points. AUC values ranged from 0.5 (not different from a randomly-selected predictive distribution) to 1 (with perfect predictive ability). Models having AUC values > 0.9 were considered to have very good, >0.8 good, and >0.7 useful discrimination abilities^69^. The final map was visualized and processed using the ArcGIS platform (http://www.esri.com/software/arcgis).

## Electronic supplementary material

supplementary materials
