# Supplementary material for: The population genetic structure of Corythucha ciliata (Say) (Hemiptera: Tingidae) provides insights into its distribution and invasiveness
Source: Sci Rep. 2017 Apr 4;7:635. doi: 10.1038/s41598-017-00279-5 (PMC5428010; doi:10.1038/s41598-017-00279-5)
Supplement: Supplementary file 1 — supplementary materials [file 41598_2017_279_MOESM1_ESM.pdf]

The population genetic structure of *Corythucha ciliata* (Say) (Hemiptera: Tingidae) provides insights into its distribution and invasiveness

Wen-Yan Yang[1], Xiao-Tian Tang[1], Rui-Ting Ju[3], Yong Zhang[4], Yu-Zhou Du[1, 2*]

1. School of Horticulture and Plant Protection & Institute of Applied Entomology, Yangzhou University, Yangzhou 225009, China
2. Jiangsu Key Laboratory of Crop Genetics and Physiology/Co-Innovation Center for Modern Production Technology of Grain Crops, Yangzhou University, Yangzhou 225009, China
3. Ministry of Education Key Laboratory for Biodiversity Science and Ecological Engineering, Fudan University, Shanghai 200438, China.
4. Department of Biology, University of Nevada, Reno, NV, USA

Corresponding author：Yu-Zhou Du, School of Horticulture and Plant Protection & Institute of Applied Entomology, Yangzhou University, 48 Wenhui Ro (East), Jiangsu, Yangzhou 225009, China; Tel: 086-514-87971854; Fax: 086-514-87347537; E-mail: yzdu@yzu.edu.cn

Supplementary Table S1. Parameters of genetic diversity of *Corythucha ciliata* based on mtDNA genes.

Gene	Population	V	n	H	π	N
COI	HF	4.0	2.0	0.467	0.00296	10
	YCC	4.0	2.0	0.200	0.00127	10
	GY	0.0	1.0	0.000	0.00000	10
	WH	0.0	1.0	0.000	0.00000	10
	HY	0.0	1.0	0.000	0.00000	10
	ZZ	0.0	1.0	0.000	0.00000	10
	CS	8.0	3.0	0.378	0.00254	10
	LY	4.0	2.0	0.356	0.00225	10
	NJ	0.0	1.0	0.000	0.00000	10
	SQ	5.0	3.0	0.511	0.00183	10
	TC	4.0	2.0	0.356	0.00225	10
	XZ	5.0	3.0	0.600	0.00201	10
	YZ	0.0	1.0	0.000	0.00000	10
	ZJ	9.0	3.0	0.711	0.00733	10
	YC	0.0	1.0	0.000	0.00000	10
	JA	1.0	2.0	0.200	0.00032	10
	CD	4.0	2.0	0.356	0.00225	10
	HZ	4.0	2.0	0.356	0.00225	10
	XA	0.0	1.0	0.000	0.00000	10
	YW	0.0	1.0	0.000	0.00000	10
	TA	4.0	2.0	0.200	0.00127	10
	SV	1.0	2.0	0.200	0.00032	10
	IT	1.0	2.0	0.533	0.00085	10
ND1	HF	0.0	1.0	0.000	0.00000	10
	YCC	1.0	2.0	0.356	0.00047	10
	GY	0.0	1.0	0.000	0.00000	10
	WH	0.0	1.0	0.000	0.00000	10
	HY	0.0	1.0	0.000	0.00000	10
	ZZ	0.0	1.0	0.000	0.00000	10
	CS	4.0	2.0	0.467	0.00249	10
	LY	0.0	1.0	0.000	0.00000	10

	NJ	3.0	2.0	0.533	0.00213	10
	SQ	1.0	2.0	0.200	0.00027	10
	TC	0.0	1.0	0.000	0.00000	10
	XZ	0.0	1.0	0.000	0.00000	10
	YZ	0.0	1.0	0.000	0.00000	10
	ZJ	3.0	2.0	0.467	0.00186	10
	YC	0.0	1.0	0.000	0.00000	10
	JA	0.0	1.0	0.000	0.00000	10
	CD	1.0	2.0	0.533	0.00071	10
	HZ	1.0	2.0	0.533	0.00071	10
	XA	1.0	2.0	0.356	0.00047	10
	YW	1.0	2.0	0.200	0.00027	10
	TA	1.0	2.0	0.356	0.00047	10
	SV	2.0	4.0	0.644	0.00098	10
	IT	0.0	1.0	0.000	0.00000	10
ND5	HF	0.0	1.0	0.000	0.00000	10
	YCC	0.0	1.0	0.000	0.00000	10
	GY	0.0	1.0	0.000	0.00000	10
	WH	0.0	1.0	0.000	0.00000	10
	HY	1.0	2.0	0.467	0.00059	10
	ZZ	1.0	2.0	0.200	0.00025	10
	CS	6.0	2.0	0.467	0.00356	10
	LY	0.0	1.0	0.000	0.00000	10
	NJ	0.0	1.0	0.000	0.00000	10
	SQ	0.0	1.0	0.000	0.00000	10
	TC	2.0	2.0	0.356	0.00090	10
	XZ	2.0	2.0	0.200	0.00051	10
	YZ	0.0	1.0	0.000	0.00000	10
	ZJ	4.0	2.0	0.533	0.00271	10
	YC	0.0	1.0	0.000	0.00000	10
	JA	0.0	1.0	0.000	0.00000	10
	CD	2.0	3.0	0.644	0.00139	10
	HZ	2.0	2.0	0.533	0.00136	10
	XA	2.0	2.0	0.356	0.00090	10

	YW	1.0	2.0	0.467	0.00059	10
	TA	2.0	2.0	0.356	0.00090	10
	SV	2.0	3.0	0.622	0.00090	10
	IT	2.0	3.0	0.511	0.00071	10
**Gene**	**Group**	**V**	**n**	**H**	**π**	**N**
**Concatenated sequences**	G1	18	19	0.611	0.00081	160
	G2	3	2	0.337	0.00047	20
	G3	8	5	0.598	0.00660	40
	G4	16	6	0.889	0.00376	10

V, variation loci per location; n, the number of haplotype per population; H, haplotype diversity; **π,** nucleotide diversity; N, the number of individuals per population or group.

Supplementary Table S2. Genetic variability and Hardy-Weinberg exact test at the microsatellites typed in *Corythucha ciliata.*

Locus	K	N	Ho	H_E	PIC	UAN	F(Null)	P-val	S.E.
CA200	20	534	0.67	0.64	0.614	2	-0.0507	0.000	0.000
CA146	11	530	0.54	0.72	0.673	2	0.0142	0.000	0.000
GT26	21	505	0.77	0.80	0.785	5	0.0220	0.000	0.000
GA70	13	540	0.55	0.75	0.721	0	0.1497	0.000	0.000
TG100	25	529	0.77	0.83	0.812	2	0.0330	0.000	0.000
CA15	10	535	0.52	0.67	0.606	2	0.1263	0.000	0.000
CA7	14	539	0.24	0.69	0.646	3	0.0490	0.000	0.000
GA365	23	494	0.57	0.74	0.732	2	0.0139	0.000	0.000
GA5	21	528	0.58	0.80	0.785	2	0.0158	0.000	0.000
Total	158								
Mean	17.5		0.58	0.74					

K, number of alleles at each locus; N, number of individuals typed for each locus; Ho, mean heterozygosity observed (direct count estimate); H_E, mean heterozygosity expected (unbiased estimate Nei, 1987); PIC, polymorphic information content; UAN, Unique allele number; F(Null), Null allele frequency estimated.

Supplementary Table S3. Characterization of nine polymorphic microsatellite loci of *Corythucha ciliate*.

Population		SSR01	SSR02	SSR03	SSR04	SSR05	SSR06	SSR07	SSR08	SSR09
HF	A	5.0000	5.0000	9.0000	5.0000	8.0000	3.0000	4.0000	4.0000	6.0000
n=24	Ae	2.8098	2.9389	4.6640	3.2960	4.5801	1.3062	1.7696	2.0209	3.1488
	Ho	1.0000	0.7391	1.0000	0.4348	0.6087	0.2174	0.2917	0.2727	0.5217
	He	0.6758	0.6744	0.8023	0.7121	0.7990	0.2396	0.4441	0.5169	0.6976
	P-HW	*	n.s.	**	**	*	n.s.	**	*	**
	N.f.	-0.2296	-0.0531	-0.1168	0.2186	0.1257	0.1590	0.2381	0.3032	0.1267
YCC	A	5.0000	3.0000	11.0000	4.0000	5.0000	3.0000	3.0000	7.0000	5.0000
n=24	Ae	1.4152	2.1333	3.5776	2.2027	3.1390	2.4615	2.3367	4.2500	4.3146
	Ho	0.2917	0.4167	0.7917	0.4167	0.6667	0.5000	0.3750	0.6471	0.7083
	He	0.2996	0.5426	0.7358	0.5576	0.6959	0.6064	0.5842	0.7879	0.7846
	P-HW	n.s.	**	n.s.	**	n.s.	n.s.	**	**	n.s.
	N.f.	0.0913	0.1146	-0.0407	0.1230	0.0263	0.0995	0.1901	0.0791	0.0533
GY	A	5.0000	3.0000	5.0000	6.0000	5.0000	3.0000	5.0000	4.0000	5.0000
n=24	Ae	2.0191	1.9459	2.3725	2.2588	2.3253	1.7430	2.8305	2.2274	2.5319
	Ho	0.3913	0.4167	0.5455	0.5000	0.6087	0.5217	0.2083	0.5652	0.5417
	He	0.5159	0.4965	0.5920	0.5691	0.5826	0.4357	0.6605	0.5633	0.6179
	P-HW	**	n.s.	n.s.	**	n.s.	n.s.	**	**	n.s.
	N.f.	0.1203	0.0865	0.0592	0.0649	-0.0314	-0.0542	0.4810	-0.0239	0.0863
WH	A	5.0000	3.0000	5.0000	6.0000	5.0000	3.0000	5.0000	4.0000	5.0000
n=24	Ae	2.0191	1.9459	2.3725	2.2588	2.3253	1.7430	2.8305	2.2274	2.5319
	Ho	0.3913	0.4167	0.5455	0.5000	0.6087	0.5217	0.2083	0.5652	0.5417
	He	0.5159	0.4965	0.5920	0.5691	0.5826	0.4357	0.6605	0.5633	0.6179
	P-HW	**	n.s.	n.s.	**	n.s.	n.s.	**	*	n.s.
	N.f.	0.1230	0.0865	0.0592	0.0649	-0.0314	-0.0542	0.4810	-0.0239	0.0863
HY	A	8.0000	4.0000	7.0000	8.0000	9.0000	3.0000	3.0000	8.0000	8.0000
n=24	Ae	3.5776	2.6544	4.5176	3.8788	6.1604	2.3367	2.3655	3.1911	3.8658
	Ho	0.8750	0.5833	0.9583	0.5833	0.9583	0.5833	0.3330	0.6250	0.7083
	He	0.7358	0.6365	0.7952	0.7580	0.8555	0.5842	0.5895	0.7012	0.7571
	P-HW	**	n.s.	n.s.	**	*	*	**	**	**

	N.f.	-0.1290	0.0462	-0.1010	0.1098	-0.0627	-0.0154	0.2553	0.0605	0.0164
ZZ	A	5.0000	3.0000	5.0000	6.0000	5.0000	3.0000	5.0000	4.0000	5.0000
n=24	Ae	2.0191	1.9459	2.3725	2.2588	2.3253	1.7430	2.8305	2.2274	2.5319
	Ho	0.3913	0.4167	0.5455	0.5000	0.6087	0.5217	0.2083	0.5652	0.5417
	He	0.5159	0.4965	0.5920	0.5691	0.5826	0.4357	0.6605	0.5633	0.6179
	P-HW	**	n.s.	n.s.	n.s.	n.s.	n.s.	**	*	n.s.
	N.f.	0.1203	0.0865	0.0592	0.0649	-0.0314	-0.0542	0.4810	-0.0239	0.0863
CS	A	5.0000	4.0000	7.0000	5.0000	5.0000	3.0000	6.0000	8.0000	6.0000
n=24	Ae	2.6667	2.4147	3.7042	2.4024	3.7921	2.8179	2.8800	4.2000	3.5000
	Ho	0.6250	0.3158	0.8750	0.0500	0.7391	0.7143	0.1250	0.5714	0.5714
	He	0.6383	0.6017	0.7456	0.5987	0.7527	0.6609	0.6667	0.7805	0.7317
	P-HW	**	*	n.s.	**	n.s.	n.s.	**	**	**
	N.f.	-0.0252	0.3036	-0.0818	0.7493	0.0050	-0.0401	0.6319	0.1382	0.1221
LY	A	6.0000	4.0000	9.0000	5.0000	7.0000	4.0000	5.0000	3.0000	6.0000
n=24	Ae	3.4804	3.1220	3.4054	2.9388	3.9724	1.8611	2.3462	2.1654	3.6993
	Ho	1.0000	0.8750	0.4762	0.6250	0.7917	0.2083	0.1667	1.0000	0.6087
	He	0.7278	0.6941	0.7236	0.6738	0.7642	0.4725	0.5860	0.5496	0.7459
	P-HW	n.s.	n.s.	**	**	n.s.	**	**	**	**
	N.f.	-0.1802	-0.1126	0.1927	0.0408	-0.0362	0.3628	0.5338	-0.2885	0.0812
NJ	A	9.0000	6.0000	7.0000	5.0000	9.0000	3.0000	4.0000	5.0000	8.0000
n=24	Ae	3.4453	2.6964	3.1154	3.0205	6.1818	2.6376	2.1879	2.5019	4.1215
	Ho	0.7143	0.6818	0.6667	0.8095	0.9048	0.7273	0.2105	0.3889	0.5714
	He	0.7271	0.6438	0.6984	0.6852	0.8711	0.6353	0.5576	0.6175	0.7758
	P-HW	n.s.	**	n.s.	n.s.	n.s.	n.s.	**	*	**
	N.f.	-0.0020	-0.0285	0.0262	-0.0821	-0.0317	-0.0569	0.4300	0.2257	0.1427
SQ	A	12.0000	5.0000	9.0000	6.0000	8.0000	4.0000	7.0000	3.0000	8.0000
n=24	Ae	5.4819	2.8918	4.3243	3.2053	4.3214	2.3050	2.6544	2.2909	3.7770
	Ho	1.0000	0.7143	1.0000	0.6818	0.5455	0.4348	0.2083	0.3330	0.4583
	He	0.8357	0.6702	0.7885	0.7040	0.7865	0.5787	0.6365	0.5772	0.7509
	P-HW	*	n.s.	n.s.	n.s.	**	n.s.	**	*	**
	N.f.	-0.1006	-0.0320	-0.1352	0.0205	0.1742	0.1504	0.4765	0.2651	0.2474

TC	A	5.0000	3.0000	11.0000	4.0000	5.0000	3.0000	3.0000	7.0000	5.0000
n=24	Ae	1.4152	2.1333	3.5776	2.2027	3.1390	2.4615	2.3367	4.2500	4.3146
	Ho	0.2917	0.4167	0.7917	0.4167	0.6667	0.5000	0.3750	0.6471	0.7083
	He	0.2996	0.5426	0.7358	0.5576	0.6959	0.6064	0.5842	0.7879	0.7846
	P-HW	n.s.	**	n.s.	**	n.s.	n.s.	**	**	n.s.
	N.f.	0.0913	0.1146	-0.0407	0.1230	0.0263	0.0995	0.1901	0.0791	0.0533
XZ	A	7.0000	5.0000	7.0000	6.0000	10.0000	4.0000	9.0000	7.0000	7.0000
n=24	Ae	5.8800	2.2422	3.8278	2.3655	4.0139	2.1294	2.7494	2.7245	3.0104
	Ho	0.9048	0.6667	1.0000	0.6250	0.7083	0.5833	0.1667	0.3684	0.2941
	He	0.8502	0.5698	0.7611	0.5895	0.7668	0.5417	0.6498	0.6501	0.6881
	P-HW	n.s.	n.s.	n.s.	**	**	n.s.	**	**	**
	N.f.	-0.0436	-0.0790	-0.1461	-0.0462	0.0212	-0.0450	0.5478	0.2998	0.3848
YZ	A	9.0000	6.0000	7.0000	5.0000	9.0000	3.0000	4.0000		8.0000
n=24	Ae	3.4453	2.6964	3.1154	3.0205	6.6818	2.6376	2.1879	2.5019	4.1215
	Ho	0.7143	0.6818	0.6667	0.8095	0.9048	0.7273	0.2105	0.3889	0.5714
	He	0.7271	0.6438	0.6984	0.6852	0.8711	0.6353	0.5576	0.6175	0.7758
	P-HW	n.s.	**	n.s.	n.s.	n.s.	n.s.	**	*	**
	N.f.	-0.0020	-0.0285	0.0262	-0.0821	-0.0317	-0.0569	0.4300	0.2257	0.1427
ZJ	A	6.0000	4.0000	6.0000	5.0000	8.0000	3.0000	6.0000	9.0000	7.0000
n=24	Ae	3.1051	3.6805	3.7524	4.0563	4.4825	2.2456	2.3900	3.3536	2.5600
	Ho	0.9583	0.7500	0.7500	0.5000	1.0000	0.5417	0.2500	0.3330	0.3330
	He	0.6924	0.7438	0.7491	0.7695	0.7934	0.5665	0.5940	0.7189	0.6223
	P-HW	n.s.	n.s.	*	**	**	n.s.	*	**	**
	N.f.	-0.1731	-0.0174	-0.0052	0.1937	-0.1280	0.0060	0.4184	0.3659	0.2960
YC	A	5.0000	3.0000	5.0000	6.0000	5.0000	3.0000	5.0000	4.0000	5.0000
n=24	Ae	2.0191	1.9459	2.3725	2.2588	2.3253	1.7430	2.8305	2.2274	2.5319
	Ho	0.3913	0.4167	0.5455	0.5000	0.6087	0.5217	0.2083	0.5652	0.5417
	He	0.5159	0.4965	0.5920	0.5691	0.5826	0.4357	0.6605	0.5633	0.6179
	P-HW	**	n.s.	n.s.	**	n.s.	n.s.	**	**	n.s.
	N.f.	0.1203	0.0865	0.0592	0.0649	-0.0314	-0.0542	0.4810	-0.0239	0.0863
JA	A	5.0000	3.0000	5.0000	6.0000	5.0000	3.0000	5.0000	4.0000	5.0000

n=24	Ae	2.0191	1.9459	2.3725	2.2588	2.3253	1.7430	2.8305	2.2274	2.5319
	Ho	0.3913	0.4167	0.5455	0.5000	0.6087	0.5217	0.2083	0.5652	0.5417
	He	0.5159	0.4965	0.5920	0.5691	0.5826	0.4357	0.6605	0.5633	0.6179
	P-HW	**	n.s.	n.s.	**	n.s.	n.s.	**	**	n.s.
	N.f.	0.1203	0.0865	0.0592	0.0649	-0.0314	-0.0542	0.4810	-0.0239	0.0863
CD	A	6.0000	4.0000	6.0000	4.0000	10.0000	5.0000	4.0000	9.0000	7.0000
n=24	Ae	2.1288	3.0490	3.2914	3.2451	5.1217	2.4100	2.8872	3.6376	3.2727
	Ho	0.5217	0.4783	0.9167	0.5417	0.9545	0.5417	0.2500	0.4545	0.7083
	He	0.5420	0.6870	0.7110	0.7066	0.8235	0.5975	0.6676	0.6353	0.7092
	P-HW	**	n.s.	n.s.	*	*	**	**	**	n.s.
	N.f.	0.0308	0.1709	-0.1380	0.1158	-0.0914	0.0404	0.4235	0.1601	-0.0164
HZ	A	6.0000	4.0000	6.0000	4.0000	10.0000	5.0000	4.0000	9.0000	7.0000
n=24	Ae	2.1288	3.0490	3.2914	3.2451	5.1217	2.4100	2.8872	3.6376	3.2727
	Ho	0.5217	0.4783	0.9167	0.5417	0.9545	0.5417	0.2500	0.4545	0.7083
	He	0.5420	0.6870	0.7110	0.7066	0.8235	0.5975	0.6676	0.6353	0.7092
	P-HW	**	n.s.	n.s.	*	*	**	**	**	n.s.
	N.f.	0.0308	0.1709	-0.1380	0.1158	-0.0914	0.0404	0.4235	0.1601	-0.0164
XA	A	6.0000	5.0000	6.0000	4.0000	7.0000	4.0000	5.0000	4.0000	9.0000
n=24	Ae	2.4935	1.9726	3.6805	2.0682	3.9588	2.7494	3.2179	3.0396	3.1947
	Ho	0.5833	0.4167	0.6250	0.4167	0.7083	0.5417	0.5000	0.9583	0.4091
	He	0.6117	0.5035	0.7438	0.5275	0.7633	0.6498	0.7039	0.6853	0.7030
	P-HW	**	**	n.s.	n.s.	n.s.	n.s.	n.s.	*	**
	N.f.	0.0307	0.0987	0.0888	0.1344	0.0299	0.0765	0.1758	-0.1714	0.2595
YW	A	8.0000	4.0000	7.0000	8.0000	9.0000	3.0000	3.0000	8.0000	8.0000
n=24	Ae	3.5776	2.6544	4.5176	3.8788	6.1604	2.3367	2.3655	3.1911	3.8658
	Ho	0.8750	0.5833	0.9583	0.5833	0.9583	0.5833	0.3330	0.6250	0.7083
	He	0.7358	0.6365	0.7952	0.7580	0.8555	0.5842	0.5895	0.7012	0.7571
	P-HW	**	n.s.	n.s.	**	**	*	**	**	**
	N.f.	-0.1290	0.0462	-0.1010	0.1098	-0.0627	-0.0154	0.2553	0.0605	0.0164
TA	A	3.0000	5.0000	6.0000	4.0000	9.0000	3.0000	5.0000	2.0000	5.0000
n=24	Ae	2.6122	3.6571	3.8278	3.5015	4.5408	2.0719	3.1304	2.0000	3.5139

	Ho	1.0000	0.7500	0.8824	0.8333	0.7826	0.6250	0.2917	1.0000	0.7619
	He	0.6303	0.7420	0.7611	0.7296	0.7971	0.5284	0.6950	0.5116	0.7329
	P-HW	**	n.s.	**	**	*	n.s.	**	**	**
	N.f.	-0.2482	-0.0213	-0.1117	-0.0792	0.0054	-0.0748	0.3948	-0.3076	-0.0195
SV	A	6.0000	6.0000	8.0000	8.0000	11.0000	5.0000	4.0000	10.0000	11.0000
n=24	Ae	2.4953	2.8365	5.4078	5.2364	5.3481	2.8140	2.9422	5.3780	4.3539
	Ho	0.8261	0.4783	0.8636	0.7083	0.9545	0.4545	0.9090	0.5714	0.6087
	He	0.6126	0.6618	0.8340	0.8262	0.8319	0.6596	0.6755	0.8339	0.7874
	P-HW	n.s.	**	**	**	**	**	**	**	**
	N.f.	-0.1815	0.1608	-0.0228	0.0663	-0.0901	0.1750	0.6945	0.1730	0.1080
IT	A	6.0000	6.0000	8.0000	8.0000	11.0000	5.0000	4.0000	10.0000	11.0000
n=24	Ae	2.4953	2.8365	5.4078	5.2364	5.3481	2.8140	2.9422	5.3780	4.3539
	Ho	0.8261	0.4783	0.8636	0.7083	0.9545	0.4545	0.9090	0.5714	0.6087
	He	0.6126	0.6618	0.8340	0.8262	0.8319	0.6596	0.6755	0.8339	0.7874
	P-HW	n.s.	**	**	**	**	**	**	**	**
	N.f.	-0.1815	0.1608	-0.0228	0.0663	-0.0901	0.1750	0.6945	0.1730	0.1080
	A	18.0000	9.0000	20.0000	11.0000	23.0000	8.0000	12.0000	21.0000	19.0000
n=24	Ae	2.7826	3.5895	5.1016	4.1021	5.8767	3.0564	3.2896	3.9559	5.1531
	Ho	0.6735	0.5463	0.7723	0.5566	0.7727	0.5243	0.2435	0.5740	0.5806
	He	0.6412	0.7221	0.8048	0.7569	0.8306	0.6734	0.6967	0.7480	0.8067
	P-HW	-	-	-	-	-	-	-	-	-
	N.f.	-	-	-	-	-	-	-	-	-

A, mean number of alleles; Ae, effective number of alleles; Ho, observed heterozygosities; H_E, expected heterozygosities; P-HW, test for Hardy-Weinberg equilibrium; n.s., denotes a significant deviation from Hardy-Weinberg equilibrium; N.F., null allele frequency. * denotes a significant deviation from Hardy-Weinberg equilibrium ($p < 0.05$); **denotes a significant deviation from Hardy-Weinberg equilibrium ($p < 0.01$)

Supplementary Table S4. Genetic diversity measures in each group of *Corythucha ciliata*.

Group	AR	A	Ho	H_E	Fis
Group I	4.56	4.67	0.48	0.56	0.11
Group II	12.16	16.11	0.61	0.76	0.20

AR, allelic richness; A, number of alleles; Ho, observed heterozygosity; H_E, expected heterozygosity; Fis, inbreeding index.

Supplementary Table S5. Pairwise F_{ST} of *Corythucha ciliata* based on concatenated mtDNA sequences and microsatellite data.

Populations	HF	YCC	GY	WH	HY	ZZ	CS	LY	NJ	SQ	TC	XZ	YZ	ZJ	YC	JA	CD	HZ	XA	YW	TA	SV	IT
HF		0.20**	0.32**	0.32**	0.22**	0.32**	0.13**	0.07**	0.10**	0.04**	0.29**	0.10**	0.10**	0.11**	0.32**	0.32**	0.20**	0.20**	0.22**	0.22**	0.09**	0.14**	0.14**
YCC	0.16		0.07**	0.07**	0.06**	0.07**	0.14**	0.23**	0.16**	0.24**	-0.02	0.23**	0.16**	0.16**	0.07**	0.07**	0.07**	0.07**	0.04**	0.06**	0.16**	0.12**	0.12**
GY	0.22	0.03		-0.02	0.11**	-0.02	0.16**	0.28**	0.19**	0.25**	0.07**	0.27**	0.19**	0.19**	-0.02	-0.02	0.05**	0.05**	0.10**	0.11**	0.19**	0.12**	0.12**
WH	0.22	0.03	0.00		0.11**	-0.02	0.16**	0.28**	0.19**	0.25**	0.07**	0.27**	0.19**	0.19**	-0.02	-0.02	0.05**	0.05**	0.10**	0.11**	0.19**	0.12**	0.12**
HY	0.22*	0.10	0.22	0.22		0.11**	0.07**	0.16**	0.09**	0.16**	0.06**	0.18**	0.095**	0.09**	0.11**	0.11**	0.04**	0.04**	0.06**	-0.02	0.11**	0.07**	0.07**
ZZ	0.21	0.03	0.00	0.00	0.17		0.16**	0.28**	0.19**	0.25**	0.07	0.27***	0.19**	0.19**	-0.02	-0.02	0.05**	0.05**	0.10**	0.11**	0.19**	0.12**	0.12**
CS	0.15	0.12	0.18	0.17**	0.14	0.15*		0.07**	0.02**	0.07**	0.14**	0.07**	0.02**	0.01**	0.1**	0.16**	0.03**	0.03**	0.10**	0.07**	0.06**	0.00**	0.00**
LY	0.77**	0.77**	0.88**	0.88**	0.83**	0.86**	0.49**		0.06**	0.02**	0.24	0.01**	0.06**	0.05**	0.28**	0.28**	0.14**	0.14**	0.18**	0.16**	0.03**	0.08**	0.08**
NJ	0.85**	0.89**	0.93**	0.93**	0.91**	0.92**	0.62**	0.88**		0.06**	0.16**	0.05**	-0.02	0.04**	0.19**	0.19**	0.07**	0.07**	0.10**	0.09**	0.05**	0.01**	0.01**
SQ	0.79**	0.79**	0.89**	0.89**	0.85**	0.88**	0.52**	-0.03	0.89**		0.24**	0.03**	0.06**	0.07**	0.25**	0.25**	0.14**	0.14**	0.18**	0.16**	0.04**	0.08**	0.08**
TC	0.16*	0.00	0.11	0.11	0.06	0.07	0.09	0.66**	0.85**	0.69**		0.23**	0.16**	0.16**	0.07**	0.07**	0.07**	0.07**	0.04**	0.06**	0.16**	0.12**	0.12**
XZ	0.77**	0.77**	0.87**	0.875**	0.83**	0.85**	0.51**	0.00	0.88**	-0.07	0.66**		0.05**	0.05**	0.27**	0.27**	0.14**	0.14**	0.18**	0.18**	0.06**	0.07**	0.07**
YZ	0.92**	0.95**	1.00**	1.00**	0.98**	0.99**	0.71**	0.94*	0.33	0.95**	0.92**	0.94**		0.04**	0.19**	0.19**	0.07**	0.07**	0.10**	0.09**	0.05**	0.01**	0.01**
ZJ	0.49**	0.50**	0.57**	0.57**	0.54**	0.56**	0.23**	0.20*	0.45**	0.23*	0.43**	0.27*	0.57**		0.19**	0.19**	0.06**	0.06**	0.08**	0.09**	0.02**	0.04**	0.04**
YC	0.22	0.03	0.00	0.00	0.22	0.00	0.18	0.88**	0.93**	0.89**	0.11	0.87**	1.00**	0.57**		-0.02	0.05**	0.05**	0.10**	0.11**	0.19**	0.12**	0.12**
JA	0.21	0.00	0.00	0.00	0.16*	0.00	0.16*	0.86**	0.93**	0.88**	0.07	0.85**	0.99**	0.56**	0.00		0.05**	0.05**	0.10**	0.11**	0.19**	0.12**	0.12**
CD	0.25**	0.12	0.27**	0.27**	0.16*	0.23**	0.06	0.49**	0.81**	0.54**	0.01	0.52**	0.88**	0.32**	0.27**	0.24**		-0.02	0.06**	0.04**	0.08**	0.01**	0.01**
HZ	0.28**	0.13	0.31**	0.31**	0.22**	0.27**	0.07	0.48**	0.81**	0.54**	0.07	0.51**	0.88**	0.31**	0.31**	0.28**	-0.09		0.06**	0.04**	0.08**	0.01**	0.01**
XA	0.21**	0.07	0.19	0.19	0.05	0.11	0.08	0.74**	0.87**	0.77**	0.03	0.75**	0.95**	0.46**	0.19	0.17	0.01*	0.05		0.06**	0.11**	0.09**	0.09**
YW	0.20**	0.05	0.17	0.17	-0.09	0.13	0.12	0.81**	0.90**	0.84**	0.05	0.81**	0.97**	0.52**	0.17	0.13	0.12**	0.17**	0.00		0.11**	0.07**	0.07**
TA	0.74*	0.72**	0.84**	0.84**	0.79**	0.82**	0.46**	0.00	0.87**	-0.02	0.60**	-0.02	0.93**	0.23*	0.84**	0.82**	0.46**	0.46	0.71**	0.78**		0.05**	0.05**
SV	0.34**	0.30**	0.41**	0.45**	0.20*	0.40**	0.11*	0.70**	0.86**	0.73**	0.16*	0.71**	0.93**	0.42**	0.451**	0.41**	0.04	0.11	0.09	0.16**	0.67**		-0.02
IT	0.45**	0.44**	0.63**	0.63**	0.38**	0.58**	0.19**	0.74**	0.88**	0.76**	0.23**	0.74**	0.95**	0.45**	0.634**	0.57**	0.13**	0.22**	0.27**	0.35**	0.70**	0.02	

F_{ST} of *C. ciliata* based on concatenated mtDNA sequences (below the diagonal); F_{ST} of *C.ciliata* based on microsatellite data (above the diagonal); *, indicated $p < 0.05$; **, indicated $p < 0.01$.

Supplementary Table S6. Estimates of gene flow between 23 *Corythucha ciliata* populations based on concatenated mtDNA sequences.

Pop. i	Θ	HF →i	YCC →i	GY →i	WH →i	HY →i	ZZ →i	CS →i	LY →i	NJ→ i	SQ →i	TC →i	XZ →i	YZ →i	ZJ→ i	YC →i	JA→ i	CD →i	HZ →i	XA →i	YW →i	TA →i	SV →i	IT- i
**HF**	0.00095		544.2	418.0	540.8	481.1	452.9	476.8	564.1	343.7	572.3	405.0	398.4	527.0	**435.4**	466.0	514.0	520.4	526.4	622.6	**595.9**	476.6	602.8	508
**YCC**	0.20230	537.5		502.5	799.4	388.7	495.4	328.3	455.1	**493.0**	338.7	592.1	639.0	**428.3**	527.2	536.9	438.2	549.6	467.6	423.8	**422.4**	335.9	399.2	549
**GY**	0.00016	636.4	664.3		**483.9**	**533.8**	515.1	777.3	**477.3**	573.7	521.2	420.6	404.2	521.2	676.5	480.4	541.8	581.1	686.0	493.4	**514.4**	422.7	**496.6**	436
**WH**	0.00505	552.8	657.7	**403.6**		523.2	476.9	**490.9**	459.1	625.4	608.7	610.7	621.6	604.3	399.3	565.5	544.2	645.0	594.8	410.4	543.3	397.9	458.2	476
**HY**	0.00822	516.1	494.0	**520.6**	613.9		476.7	543.8	335.0	363.5	529.2	400.1	420.2	567.8	578.0	331.4	386.5	466.3	569.7	547.0	538.3	**510.1**	457.2	512
**ZZ**	0.00020	475.1	548.7	568.3	577.8	558.4		508.5	402.9	489.5	457.9	437.3	477.3	544.7	427.6	481.8	334.8	569.8	543.9	494.7	497.5	437.8	579.2	477
**CS**	0.02182	516.9	510.6	393.4	**543.4**	324.1	545.4		606.6	**541.5**	718.1	436.2	580.5	485.5	515.7	417.2	561.3	451.5	547.1	477.8	**427.1**	381.2	483.8	479
**LY**	0.00015	361.9	351.3	**598.1**	467.1	400.9	688.3	324.9		485.1	693.6	611.5	463.8	509.4	552.4	**507.6**	571.0	498.3	359.6	599.6	561.0	642.8	680.4	563
**NJ**	0.00017	440.7	**500.5**	407.9	349.1	501.2	600.1	**676.5**	441.7		455.6	627.9	575.8	611.3	471.8	385.3	355.1	548.6	324.3	545.0	497.5	496.6	418.1	491
**SQ**	0.00065	408.5	479.6	352.7	509.0	375.9	460.6	534.9	421.5	540.2		569.0	490.2	383.1	608.4	367.8	353.6	515.2	407.3	465.3	**461.4**	382.3	345.2	427
**TC**	0.03082	470.2	513.4	447.7	360.1	517.5	377.8	471.7	640.4	530.1	567.3		415.7	590.7	532.3	520.4	529.9	570.5	516.2	653.5	546.9	**410.9**	655.4	480
**XZ**	0.00061	555.6	486.7	503.0	611.1	406.5	389.5	459.2	587.6	604.3	472.7	426.6		447.6	556.7	390.1	528.6	557.3	480.4	431.2	409.9	**515.5**	600.5	361
**YZ**	0.00009	543.8	**556.9**	611.5	438.7	453.7	610.2	382.3	435.8	538.3	380.5	274.7	554.5		692.9	389.4	366.9	523.3	**545.5**	577.7	412.5	506.4	**472.3**	573
**ZJ**	0.04176	**409.8**	542.3	556.9	586.5	473.1	282.0	514.8	619.4	459.0	288.9	465.8	560.5	592.9		442.0	563.5	576.3	261.4	553.5	633.8	546.5	433.2	465
**YC**	0.00017	438.5	509.9	472.6	631.8	458.6	695.5	568.4	**575.9**	456.0	648.7	683.1	370.6	423.0	390.9		488.4	772.6	534.5	540.5	499.8	599.2	491.1	433
**JA**	0.00044	545.5	605.5	658.4	620.1	495.6	494.5	466.2	659.4	527.6	528.5	609.0	360.3	534.1	492.3	505.4		**678.0**	471.7	452.6	536.4	443.9	389.1	579
**CD**	0.03080	490.2	465.4	713.3	372.1	472.1	564.7	567.4	570.4	632.6	555.1	607.9	669.3	555.8	387.2	474.6	**447.5**		385.0	371.3	639.3	**458.8**	608.5	438
**HZ**	0.00281	489.4	549.5	479.7	442.0	613.9	488.1	649.8	553.0	586.9	609.6	711.6	484.3	**462.0**	448.7	488.9	424.1	571.4		**442.7**	537.2	620.1	621.5	524
**XA**	0.02551	605.4	508.1	588.7	489.6	613.9	501.1	678.5	469.8	465.7	481.5	399.9	519.0	394.1	435.7	423.0	554.4	490.7	**528.0**		458.1	710.9	579.0	600
**YW**	0.01316	**492.5**	**472.4**	443.7	683.7	295.9	484.4	**489.6**	483.4	533.9	**532.4**	628.0	550.0	568.7	441.6	487.3	314.2	648.2	628.1	577.0		501.3	461.8	469
**TA**	0.00081	457.2	438.5	364.1	545.0	**480.0**	516.6	335.0	451.0	621.6	558.3	**566.1**	**557.2**	461.9	512.0	542.6	687.3	**577.8**	412.1	673.0	418.7		498.1	317
**SV**	0.02314	540.1	421.5	529.0	653.6	617.4	547.6	591.3	638.6	372.6	500.5	485.4	578.0	**401.3**	248.9	435.8	541.0	593.2	559.9	611.5	601.5	551.3		312
**IT**	0.00037	490.0	439.7	510.3	336.1	611.3	575.1	516.5	426.0	465.4	358.2	603.2	436.1	294.1	560.7	455.0	358.9	548.2	424.0	579.3	638.8	654.3	672.0	

Pop., populations; Θ, the effective mutation scaled population size; the bidirectional mutation-scaled immigration rate (M) was estimated for 253 population pairs. Instances of asymmetrical gene flow are indicated in bold. The source population is indicated in columns, the target population in row.

Supplementary Table S7. Estimates of gene flow between 23 *Corythucha ciliata* populations based on microsatellite data.

Pop. i	Θ	HF →i	YCC →i	GY →i	WH →i	HY →i	ZZ →i	CS →i	LY →i	NJ→ i	SQ →i	TC →i	XZ →i	YZ →i	ZJ→ i	YC →i	JA→ i	CD →i	HZ →i	XA →i	YW →i	TA →i	SV →i	IT- i
**HF**	0.05770		749.0	698.5	442.3	299.3	764.3	274.7	652.7	668.5	415.9	549.8	750.0	531.9	**728.3**	668.3	641.5	618.7	936.6	632.3	**499.3**	753.6	308.4	238
**YCC**	0.01922	650.1		606.2	366.1	354.3	237.1	684.4	362.7	**700.5**	317.3	490.5	549.2	**468.4**	557.4	559.5	304.5	248.7	350.6	457.1	**717.1**	696.9	442.8	665
**GY**	0.05044	434.8	633.8		**129.0**	**723.7**	206.1	552.9	**249.1**	487.8	534.8	697.5	357.9	234.5	186.1	579.8	550.2	697.6	476.7	497.7	**539.3**	550.4	**433.0**	645
**WH**	0.06404	680.6	720.2	**256.7**		752.0	509.2	**501.2**	683.3	699.8	371.6	644.0	522.5	763.7	625.6	358.4	802.4	631.3	561.5	667.2	288.9	436.5	441.0	543
**HY**	0.03752	432.7	546.7	**585.9**	274.6		465.8	628.0	649.9	199.4	498.7	599.7	663.5	544.2	692.0	370.3	507.2	364.5	414.3	732.3	405.3	**452.7**	496.4	461
**ZZ**	0.01744	327.3	723.2	403.4	268.3	582.0		581.3	335.4	349.8	524.1	531.4	530.9	482.7	406.5	544.8	398.2	231.6	368.3	833.0	229.3	452.5	367.3	691
**CS**	0.03521	419.2	243.7	131.9	**522.9**	706.0	667.6		447.6	**543.4**	438.2	407.6	226.2	296.9	453.9	411.6	520.1	597.3	305.4	626.6	**585.4**	372.3	614.8	698
**LY**	0.04670	561.2	768.7	**143.9**	509.0	721.4	571.3	414.1		714.2	232.4	484.7	340.7	257.0	466.0	**836.2**	544.1	586.9	539.0	589.3	727.2	502.2	652.6	601
**NJ**	0.04923	568.4	**613.7**	714.8	672.9	503.1	245.9	**459.7**	455.3		265.2	535.6	801.1	432.6	172.2	518.1	621.6	557.2	502.1	609.0	576.6	503.8	422.3	669
**SQ**	0.04576	504.6	481.8	235.0	627.9	147.7	196.2	758.0	720.9	687.2		379.9	524.2	788.9	587.1	332.0	765.4	559.0	469.7	400.7	**161.8**	816.0	744.6	561
**TC**	0.03696	218.9	500.7	634.4	291.0	546.5	614.6	532.8	665.5	408.5	394.7		216.5	497.5	609.2	466.7	844.8	453.0	290.8	522.1	191.9	**683.3**	402.7	643
**XZ**	0.05714	526.8	525.1	393.9	268.3	628.9	527.5	328.2	581.2	585.8	236.0	763.0		288.9	505.2	415.4	393.7	850.7	345.0	258.5	564.4	**541.7**	355.9	448
**YZ**	0.05247	379.9	**466.3**	389.1	192.4	548.9	607.2	576.0	362.1	495.4	563.2	690.9	592.3		189.1	222.6	431.9	597.7	**486.9**	748.4	666.4	349.3	**435.2**	726
**ZJ**	0.06535	**820.6**	596.7	474.0	831.9	411.2	435.8	349.9	375.2	658.1	424.0	366.0	277.7	455.4		586.1	580.2	671.0	594.1	485.8	172.9	332.4	618.2	568
**YC**	0.01288	375.4	246.6	744.9	383.1	667.1	278.8	394.5	**658.4**	501.9	603.6	734.4	336.5	569.0	593.4		592.2	499.6	473.4	371.2	592.6	451.6	597.7	502
**JA**	0.03627	571.0	524.6	635.7	411.6	685.6	776.1	233.2	681.4	595.4	197.3	304.7	530.2	593.4	388.0	698.6		**691.8**	269.4	709.3	456.3	500.3	754.2	415
**CD**	0.04281	529.2	380.6	457.6	324.9	387.0	601.4	148.2	550.2	444.3	672.2	491.6	396.7	548.3	719.5	768.0	**524.9**		167.2	614.6	772.4	**548.2**	519.0	131
**HZ**	0.02932	607.2	503.2	682.2	476.9	235.2	441.5	292.7	256.3	471.6	610.1	450.0	586.6	**304.5**	452.2	429.7	836.9	530.2		**229.2**	673.1	534.7	508.1	548
**XA**	0.01116	304.3	765.6	578.9	277.8	345.8	475.3	307.0	299.8	633.3	303.2	443.7	594.6	676.3	679.1	380.6	647.4	635.3	**403.1**		426.4	329.0	541.6	622
**YW**	0.04231	**888.4**	**857.1**	501.2	396.8	696.1	582.0	**454.6**	351.4	353.6	**665.6**	628.2	144.0	432.6	707.1	799.4	132.0	592.8	531.1	251.3		299.5	634.4	610
**TA**	0.07573	578.5	750.0	487.8	246.6	**585.3**	750.5	367.4	825.9	262.3	585.8	**354.3**	**456.4**	259.1	615.0	810.7	153.6	**429.3**	869.4	327.4	391.3		596.2	549
**SV**	0.04605	235.5	772.7	600.3	763.3	649.0	354.4	380.8	517.9	735.5	774.2	334.2	390.3	**413.5**	471.4	300.9	473.5	512.4	736.0	245.6	414.4	431.1		585
**IT**	0.02720	356.5	428.5	479.5	266.6	466.6	280.1	396.2	814.9	513.9	317.1	587.2	570.0	585.7	448.4	301.3	856.5	604.0	632.3	371.9	253.6	579.1	454.1	

Pop., populations; Θ, the effective mutation scaled population size; the bidirectional mutation-scaled immigration rate (M) was estimated for 253 population pairs. Instances of asymmetrical gene flow are indicated in bold. The source population is indicated in columns, the target population in row.
